# Transition-Metal-Catalyzed Diarylation of Isocyanides with Triarylbismuthines for the Selective Synthesis of Imine Derivatives

**DOI:** 10.3390/ma14154271

**Published:** 2021-07-30

**Authors:** Shintaro Kodama, Yuki Yamamoto, Yohsuke Kobiki, Hitomi Matsubara, Cong Chi Tran, Shin-ichi Kawaguchi, Akihiro Nomoto, Akiya Ogawa

**Affiliations:** 1 Department of Applied Chemistry, Graduate School of Engineering, Osaka Prefecture University, 1-1 Gakuen-cho, Nakaku, Sakai, Osaka 599-8531, Japan; skodama@chem.osakafu-u.ac.jp (S.K.); syb02137@edu.osakafu-u.ac.jp (Y.Y.); yohsuke.kobiki@gmail.com (Y.K.); sv108059@edu.osakafu-u.ac.jp (H.M.); mz105131@edu.osakafu-u.ac.jp (C.C.T.); nomoto@chem.osakafu-u.ac.jp (A.N.); 2 Center for Education and Research in Agricultural Innovation, Faculty of Agriculture, Saga University, 152-1 Shonan-cho, Karatsu, Saga 847-0021, Japan

**Keywords:** arylation, isocyanide, imine, triarylbismuthine, 2,3-diarylquinoxaline

## Abstract

The transition-metal-catalyzed diarylation of isocyanides with triarylbismuthines was investigated in detail, and rhodium catalysts such as [RhCl(nbd)]_2_ were found to selectively afford *N*-alkyl diaryl ketimines. On the other hand, palladium-catalyzed diarylation proceeded with the incorporation of two molecules of isocyanide, preferentially yielding *N*,*N’*-dialkyl or *N*,*N’*-diaryl *α*-diimines. In addition, a cascade synthesis of 2,3-diarylquinoxalines starting from the palladium-catalyzed diarylation of isocyanides with triarylbismuthines was successfully achieved.

## 1. Introduction

As the chemistry of heteroatom-containing compounds has significantly grown in recent decades, the properties and reactivities of high-period elements have gradually attracted more attention [1,2,3,4,5,6,7,8,9,10,11,12]. Bismuth is the heaviest of the group 15 elements, and its organic and inorganic compounds are regarded to be nontoxic [13]. However, organobismuth compounds are generally unstable due to the weakness of the carbon–bismuth bond. An exception is triarylbismuthines (BiAr_3_, **1**), which are stable and some of them are commercially available. Therefore, synthetic applications of triarylbismuthines [14,15,16] have been investigated by many organic chemists [17,18,19,20,21,22,23,24,25] and *N*-, *O*-, *S*- and *C*-arylation reactions have been developed using triarylbismuthines as arylating reagents.

**Scheme 1:**
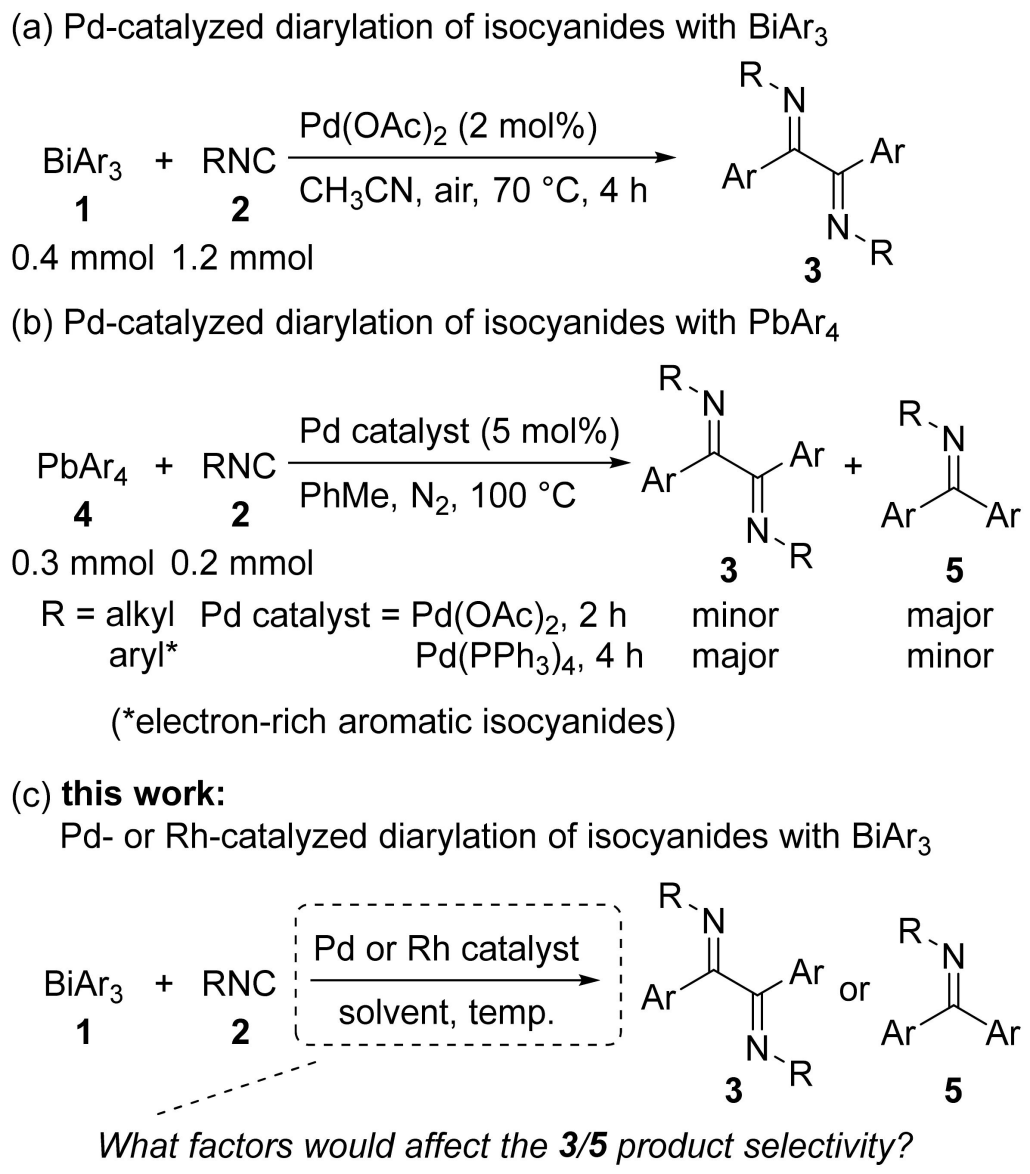
Transition-metal-catalyzed diarylation of isocyanides.

Recently, we developed novel palladium-catalyzed diarylation reactions of isocyanides **2** [26,27,28,29,30,31,32,33,34,35,36,37,38,39,40,41] using triarylbismuthines **1** and tetraarylleads **4** (Scheme 1). The use of BiAr_3_ **1** result in *α*-diimines **3** being selectively obtained (Scheme 1a) [42]. Using PbAr_4_ **4** instead of **1** led to the formation of *α*-diimines **3** and/or ketimines **5** (Scheme 1b) [43]. With aliphatic isocyanides **2** (R = alkyl), *N*-alkyl diaryl ketimines **5** were preferentially obtained, whereas *N*,*N’*-diaryl α-diimines **3** were formed when electron-rich aromatic isocyanides **2** (R = electron-rich Ar) were used.

However, it is unclear what factors would affect the product selectivity of α-diimines **3** and/or ketimines **5** using BiAr_3_. Hence, we investigated the transition-metal-catalyzed diarylation of isocyanides with BiAr_3_ **1** under several reaction conditions for the selective synthesis of imine derivatives (**3** or **5**) (Scheme 1c).

## 2. Results and Discussion

In our previous paper [42], we reported that Pd(OAc)_2_-catalyzed diarylation of isocyanides **2** with BiAr_3_ **1** selectively afforded *α*-diimines **3** (a representative result is shown in Table 1, entry 1). When the catalyst was changed to Pd(PPh_3_)_4_, a typical zero-valent palladium complex, the yield of **3aa** decreased significantly (entry 2). On the other hand, other divalent palladium complexes such as PdCl_2_ and Pd(PPh_3_)_2_Cl_2_ selectively afforded **3aa** with good yields (entries 3 and 4). Addition of PPh_3_ to Pd(OAc)_2_ resulted in lower yield of **3aa** (entry 5). The zero-valent Pd complex, Pd_2_(dba)_3_, gave **3aa** in moderate yield (entry 6). In the absence of a catalyst, barely any diarylation occurred (entry 7).

The reaction conditions for the Pd(OAc)_2_-catalyzed diarylation of **2a** with **1a** were also investigated in more detail (entries 8–18). In all cases, *α*-diimine **3aa** was obtained as the major product, mostly along with very small amounts of ketimine **5aa**. Reducing the loading of **1a** resulted in a decrease in the yield of **3aa** (entry 8). The presence of air did not inhibit the formation of **3aa** (entry 9). When the reaction was conducted at room temperature, the yield of **3aa** decreased (entry 10). Decreasing the amount of Pd(OAc)_2_ resulted in a gradual decrease in the yield of **3aa** (entries 11 and 12). Among the solvents examined (entries 13–16), acetonitrile gave the best result, obtaining **3aa** in 84% yield (entry 15). The present diarylation also proceeded even in a shorter time (4 h), affording **3aa** in 81% yield (entry 17). A similar result was also obtained under Ar atmosphere as under air (entry 18 vs. 9).

Surprisingly, changing the catalyst to rhodium complexes selectively afforded ketimine **5aa** without formation of **3aa** (Table 2, entries 1–4). For example, the diarylation using [RhCl(nbd)]_2_ exclusively afforded **5aa** in 50% yield (entry 1). Reducing the loading of this catalyst resulted in a lower yield of **5aa** (entry 2). In addition, use of excess **1a** improved the yield of **5aa** with this catalyst (entry 3). RhH(CO)(PPh_3_)_3_, which is an active catalyst for hydroformylation, was ineffective when used for the present diarylation of isocyanide **2a** (entry 4).

Since several rhodium complexes exhibited good ketimine selectivity, we next investigated the rhodium-catalyzed diarylation of *tert*-butyl isocyanide **2a** with triphenylbismuthine **1a**. The Rh-catalyzed diarylation was performed on a 0.4 mmol scale (**1a** and **2a**) using [RhCl(nbd)]_2_, and the desired ketimine **5aa** was obtained in 51% yield by adding (*p*-MeO-C_6_H_4_)_3_P as the ligand (entry 5). The diarylation using 1.0 mL of C_6_H_6_ improved the yield of **5aa** to 71% yield (entry 7). Use of 0.6 mmol of **1a** resulted in a slightly lower yield of **5aa** (entry 9 vs. 7). Some other rhodium catalysts, such as RhCl(PPh_3_)_3_, RhH(PPh_3_)_3_, RhBr(PPh_3_)_3_, [Rh(dppp)(cod)]^+^BF_4_^−^, RhCl_3_, and [Rh(OAc)_2_]_2_, were ineffective for the desired diarylation (entries 10, 11, 12, and 14–16). In addition, the diarylation failed when using other transition-metal catalysts, such as [Ru(NH_3_)_5_Cl]Cl_2_, RuCl_3_•nH_2_O, [Ir(cod)Cl]_2_, Ir(CO)Cl[*n*-C_10_F_21_]PPh_2_]_2_, Ir(CO)Cl(PPh_3_)_2_, CuI, CuCl_2_, and CoCl(PPh_3_)_3_, with no reaction taking place in most cases (these data are not shown in Table 2). Among the catalysts examined, *trans*-RhCl(CO)(PPh_3_)_3_ exhibited a moderate catalytic activity for the diarylation to give **5aa** (entry 13). Overall, when mononuclear Rh complexes were used as catalysts, many byproducts were formed via polymerization of *tert*-butyl isocyanide. In contrast, this polymerization was suppressed by using [RhCl(nbd)]_2_. These results suggest that the choice of catalysts is very important for the selective reaction between isocyanides and BiAr_3_.

**Scheme 2:**
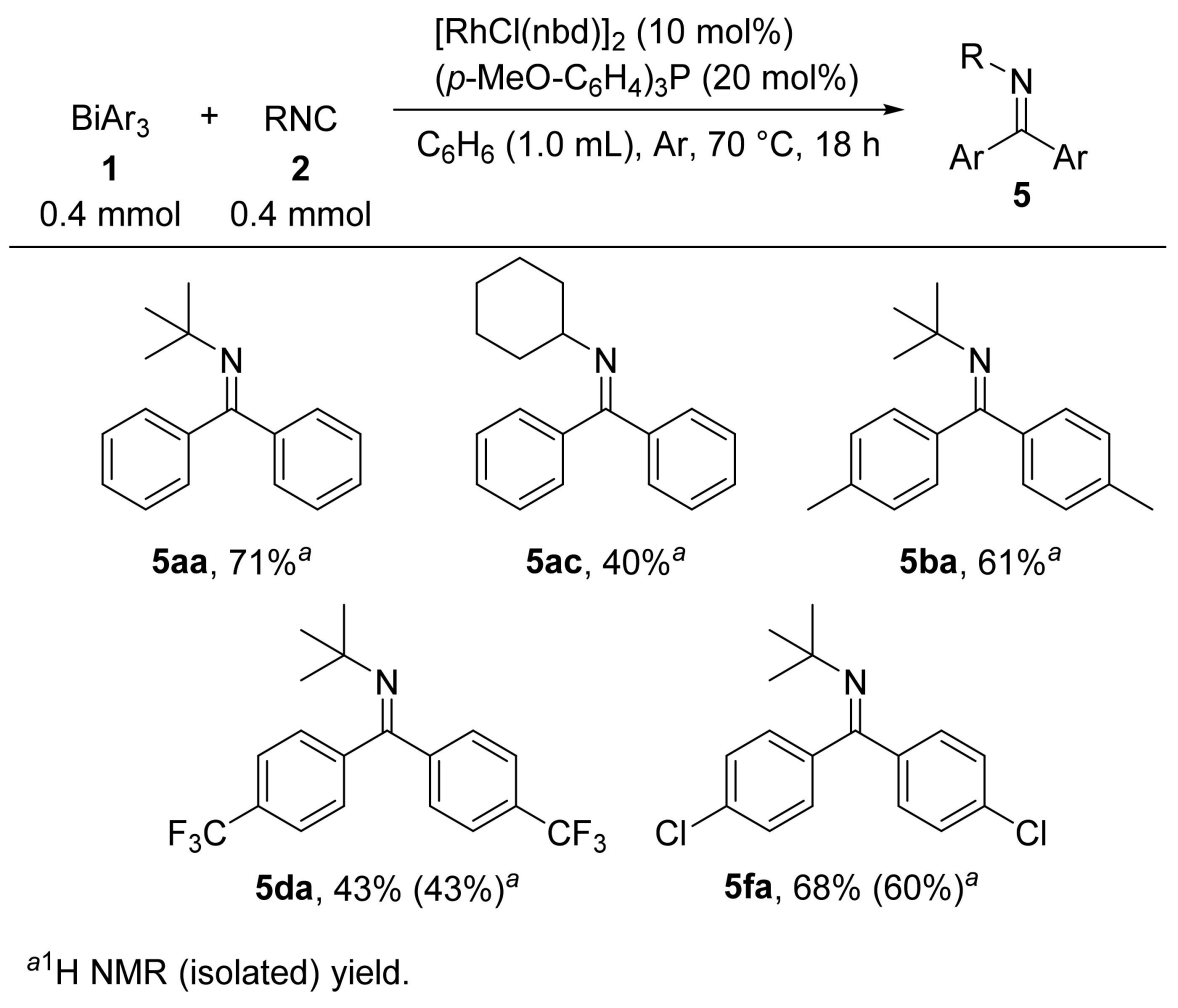
Substrate scope of the Rh-catalyzed diarylation of aliphatic isocyanides **2** with BiAr_3_ **1**.

With the optimal reaction conditions in hand (Table 2, entry 7), the scope of the Rh-catalyzed diarylation of aliphatic isocyanides **2** with BiAr_3_ **1** was examined (Scheme 2). The reaction of *t*-BuNC **2a** with BiPh_3_ **1a** afforded **5aa** in 71% yield, whereas cyclohexyl isocyanide **2c** underwent the diarylation to provide **5ac** in 40% yield. The reactions of *t*-BuNC **2a** with *p*-Me, *p*-CF_3_, and *p*-Cl-substituted triarylbismuthines **1** also proceeded to give the corresponding *N*-*tert*-butyl diaryl ketimines (**5ba**, **5da**, and **5fa**) in moderate to good yields (see Materials and Methods).

**Scheme 3:**
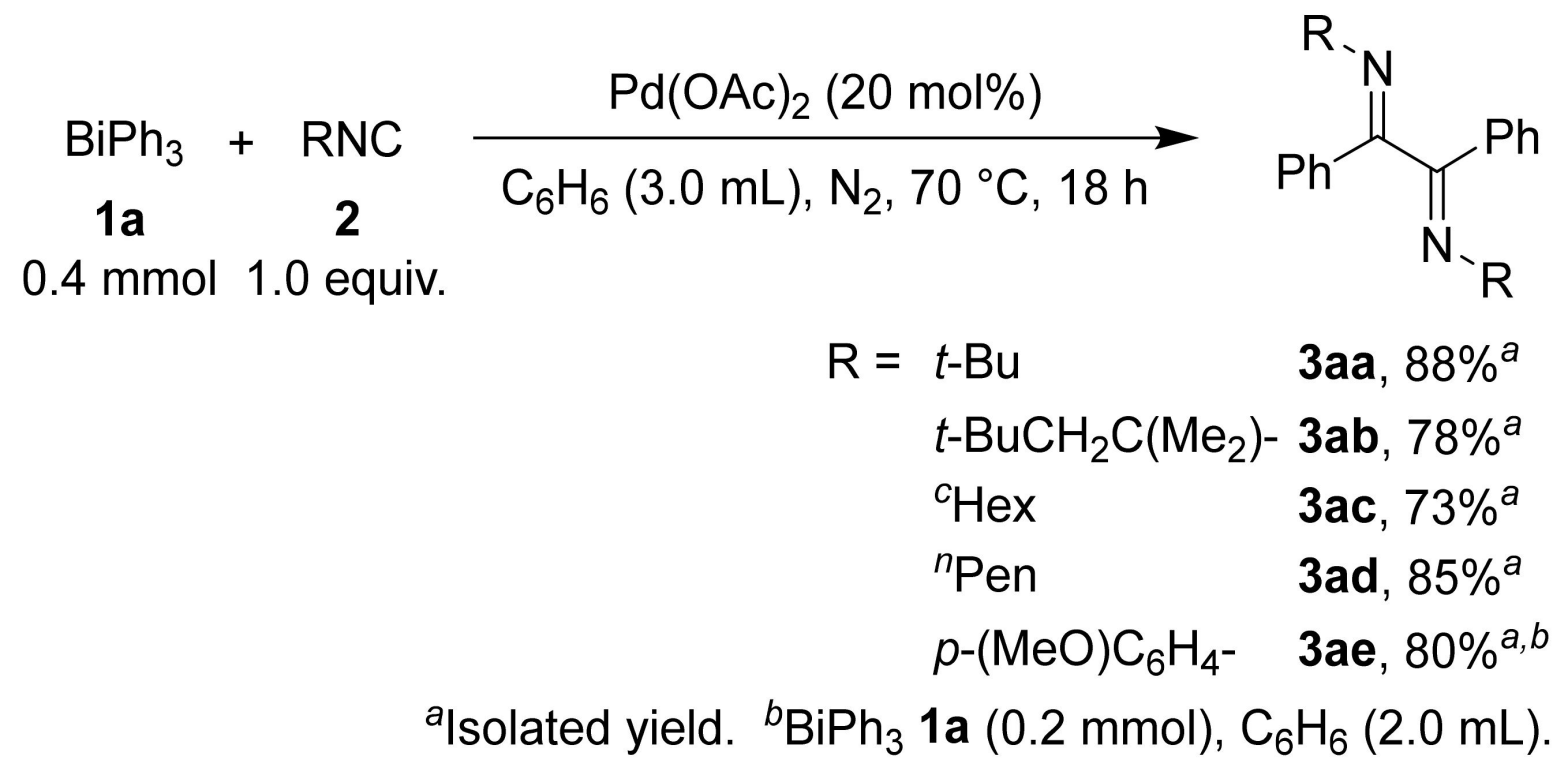
Substrate scope of the Pd-catalyzed diarylation of isocyanides **2** with BiPh_3_ **1a**.

As mentioned above, the Pd and Rh catalysts were found to afford *α*-diimines **3** and ketimines **5**, respectively, with excellent product selectivity. Conceivably, *α*-diimines **3** might be more important than ketimines **5** as synthetic intermediates. Hence, we examined the scope and limitations of this catalytic diarylation using the reaction conditions found in entry 1 of Table 1 (Scheme 3). In the cases of aliphatic isocyanides **2a**–**2d**, the corresponding *N*,*N’*-dialkyl *α*-diimines **3aa**–**3ad** were successfully obtained in good yields. The diarylation of electron-rich aromatic isocyanide **2e** also afforded the corresponding *N*,*N’*-diaryl *α*-diimine **3ae** in good yield, whereas aromatic isocyanides with electron-withdrawing groups such as *p*-nitro and *p*-cyano groups resulted in the formation of a complex mixture.

**Scheme 4:**
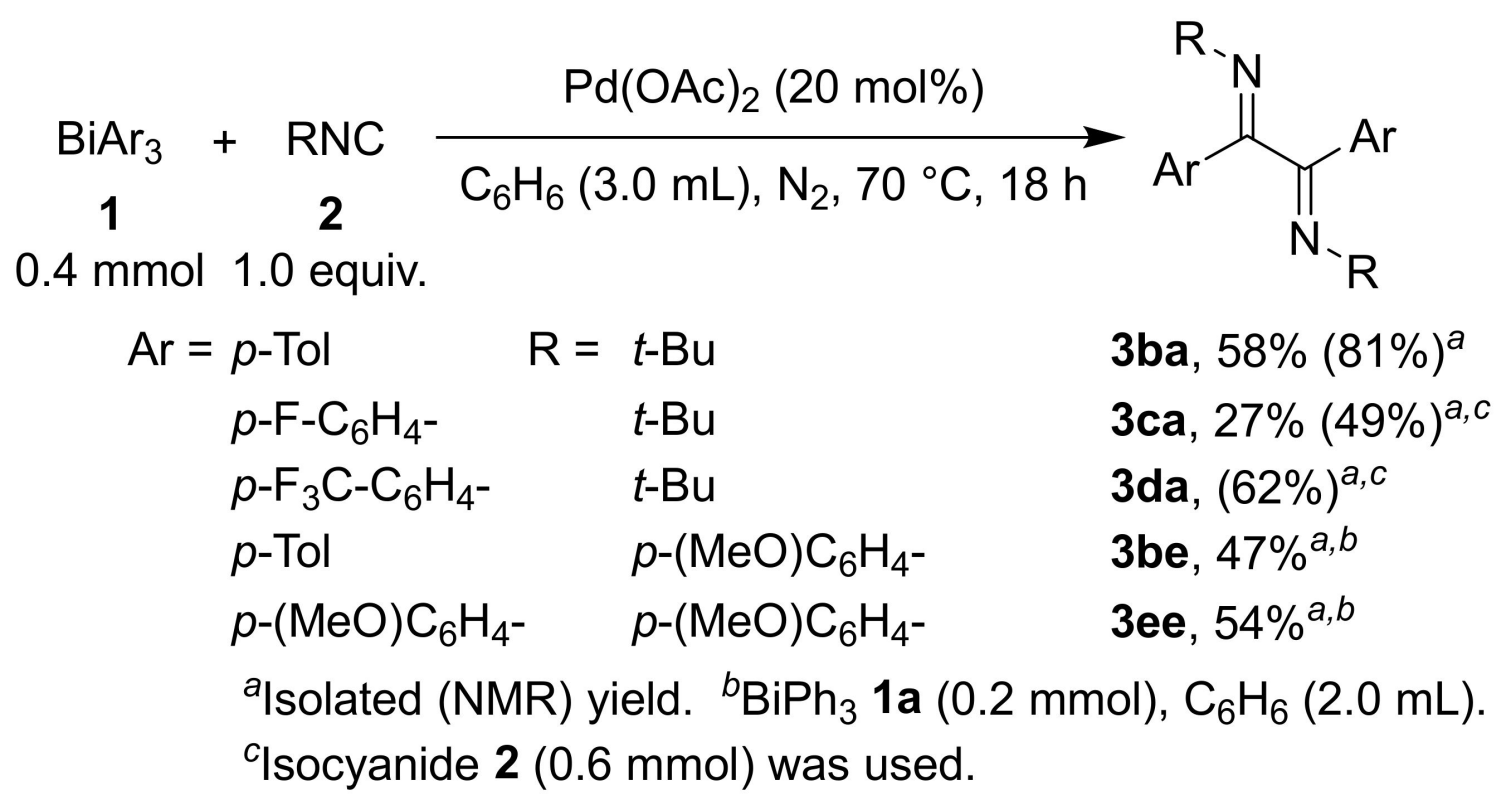
Substrate scope of the Pd-catalyzed diarylation of isocyanides **2** with BiAr_3_ **1**.

In addition, the scope and limitations of the triarylbismuthines were investigated (Scheme 4). The diarylation of *t*-BuNC **2a**, with BiAr_3_ **1b**–**1d** was conducted, and the corresponding *N*,*N’*-di*-tert*-butyl α-diimines **3ba**–**3da** were formed in moderate yields. When the *p*-methoxyphenyl isocyanide **2e** was used for the arylation, similar results were observed.

The Pd(OAc)_2_-catalyzed diarylation of *t*-BuNC **2a** with Bi(C_6_H_4_-F-*p*)_3_ **1c** was carried out under several different conditions to explore the reason for the lower yield of **3ca** (Table 3).

An equimolar reaction of **2a** with **1c** was found to afford ketimine **5ca** in parallel to *α*-diimine **3ca** (entry 1), while decreasing the amount of **1c** resulted in the selective formation of **3ca** (entry 3). Therefore, the molar ratio of **1c** to **2a** was an important factor for the selective synthesis of *α*-diimine **3ca**.

The impact of reducing the catalyst loading was then examined to allow for the easy isolation of *α*-diimines **3** (Table 4). The catalytic diarylation of isocyanide **2a** was conducted using 5 mol% Pd(OAc)_2_ and one equivalent of triphenylbismuthine **1a** to **2a** under an atmosphere of N_2_, and *α*-diimine **3aa** was obtained in low yield (entry 1). Interestingly, under an atmosphere of air, the yield of **3aa** was dramatically improved (entry 2). Using molecular oxygen instead of air was also effective (entry 3). However, the combination of divalent copper salts was ineffective for the diarylation (entries 4 and 5). Moreover, the effect of reducing the amount of BiPh_3_ **1a** was examined (entries 6–8). Even when using 1/3 equivalent of **1a**, *α*-diimine **3aa** was obtained in satisfactory yield (entry 6). This clearly indicates that all three phenyl groups on **1a** could be used for the formation of **3aa**. When the loading of Pd(OAc)_2_ was reduced to 1 mol%, the yield of **3aa** slightly decreased (entry 7). However, the use of 2 mol% of Pd(OAc)_2_ led to the formation of **3aa** in a satisfactory yield (81%). As can be seen from the data in Table 4, the use of a combination of Pd(OAc)_2_ and air reduced the loading of both catalyst and triarylbismuthine. Further examination of reaction conditions for the Pd(OAc)_2_-catalyzed diarylation in air revealed that acetonitrile was the best for the present diarylation [42].

**Scheme 5:**
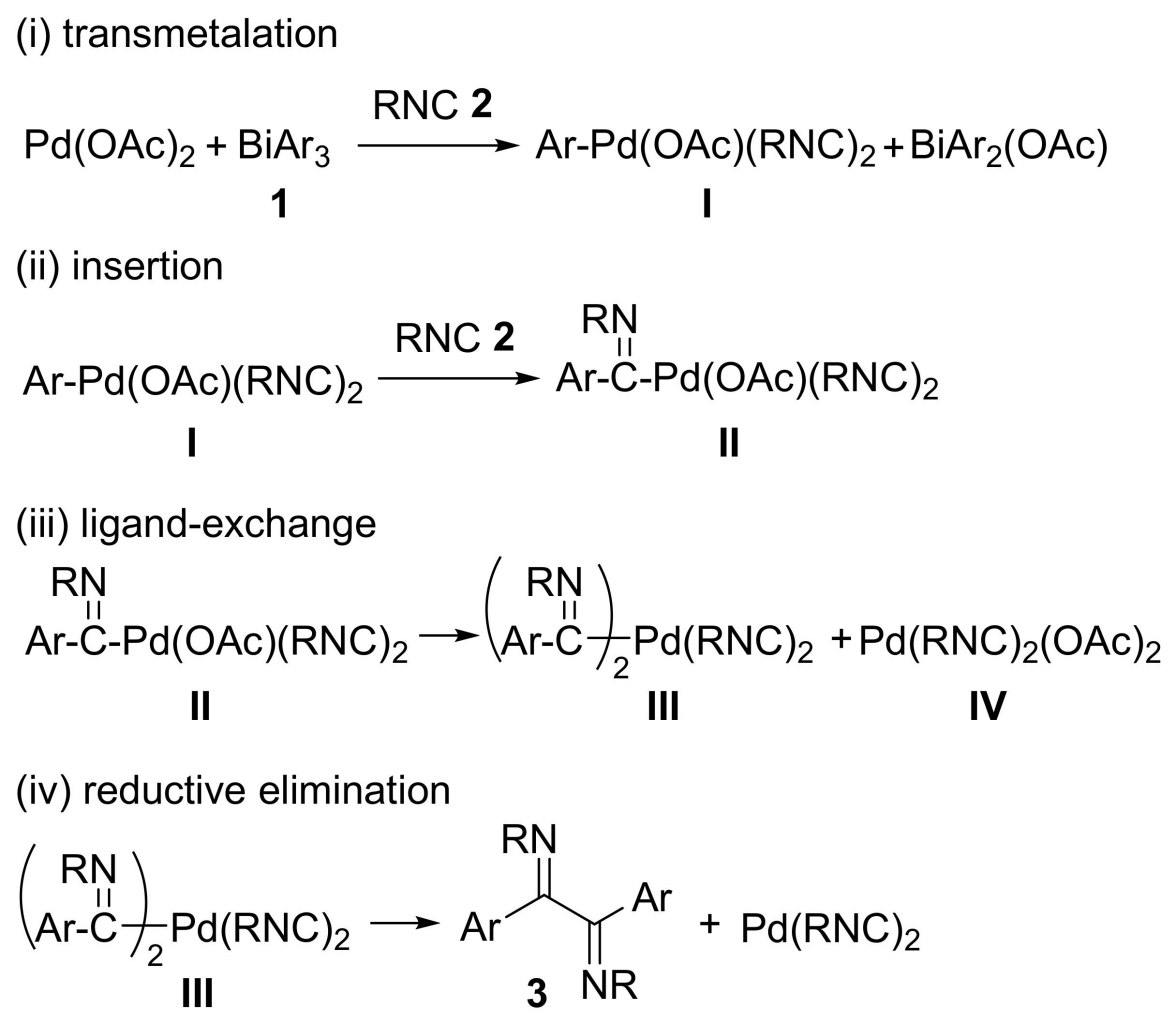
A possible pathway for Pd-catalyzed diarylation.

In our previous paper, we proposed a possible pathway for the Pd(OAc)_2_-catalyzed diarylation of isocyanide **2** with triarylbismuthine **1** to afford *α*-diimine **3**, the essence of which is shown in Scheme 5.

Transmetalation between Pd(OAc)_2_ and BiAr_3_ **1** might generate arylpalladium species **I**, into which isocyanide **2** inserts to form the imidoylpalladium species **II**. The subsequent ligand-exchange reaction of **II** with itself then leads to the palladium complexes **III** and **IV**. Reductive elimination from **III** affords the α-diimines **3** along with the Pd(0) species. Since the Pd(OAc)_2_-catalyzed diarylation proceeds smoothly in the presence of oxidizing agents such as air, the Pd(0) species might be oxidized to the Pd(II) species by air, or by the bismuth compounds present in the reaction system.

In the case of rhodium catalysts such as [RhCl(nbd)]_2_, oxidative addition of BiAr_3_ **1**, followed by insertion of isocyanide **2** into the Rh–Ar bond results in the formation of an imidoylrhodium species. Presumably, the ligand-exchange reaction is less important for the imidoylrhodium species compared with the Pd(OAc)_2_-based system. Accordingly, transmetalation of imidoylrhodium species with BiAr_3_ **1** might generate aryl imidoylrhodium species of the type “ArC(=NR)–RhL*_n_*–Ar,” and the subsequent reductive elimination might selectively afford ketimines **5**.

**Scheme 6:**
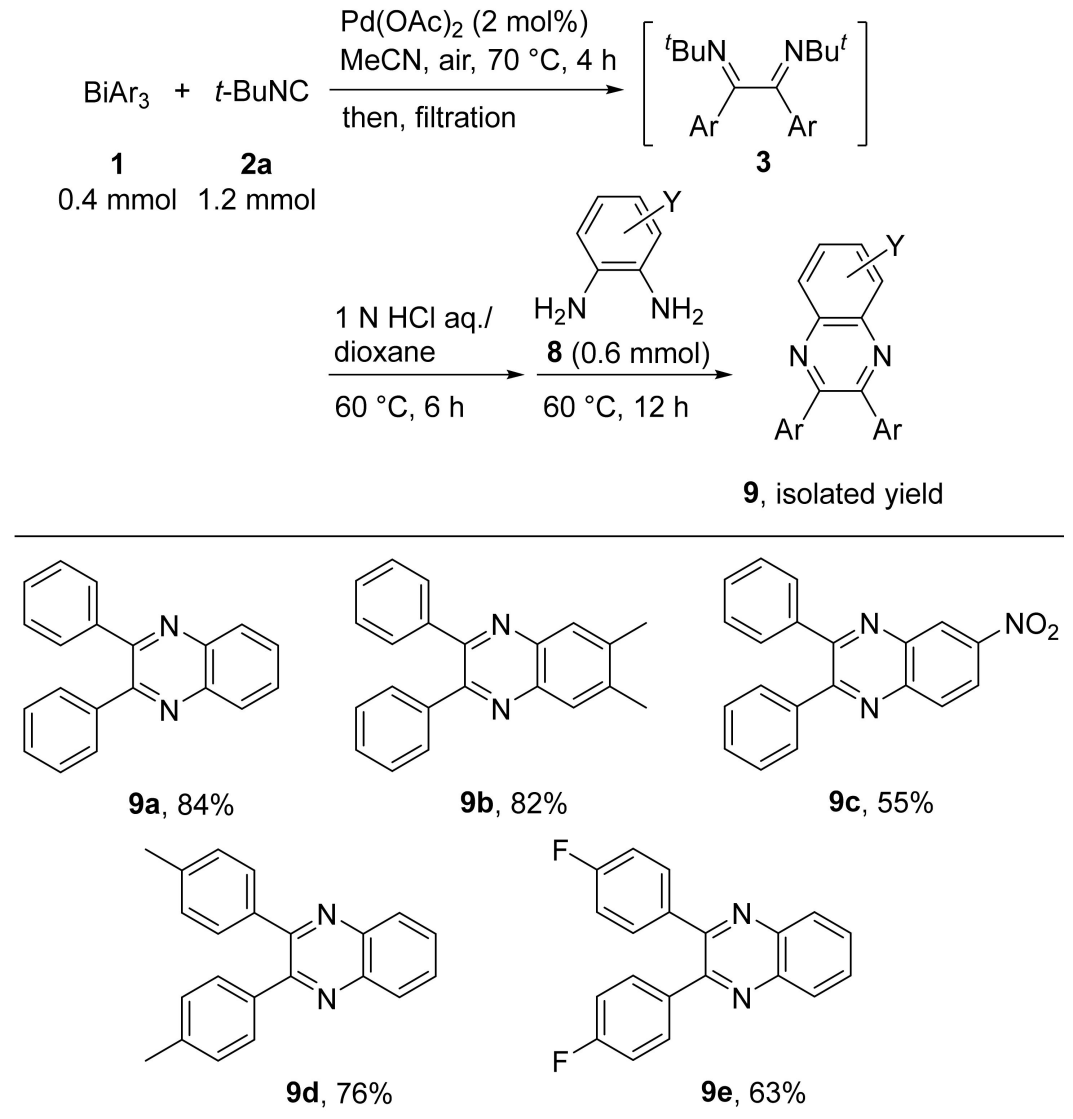
Application to cascade synthesis of 2,3-diarylquinoxalines.

Since *α*-diimines **3** are expected to be important precursors for the synthesis of nitrogen-containing heterocyclic compounds, an attempt was also made to synthesize nitrogen-containing heterocycles without purification of *α*-diimines **3** prepared by the Pd(OAc)_2_-catalyzed diarylation of isocyanides with triarylbismuthines. Hence, we next examined the synthesis of quinoxaline derivatives (Scheme 6).

After the catalytic diarylation of *t*-BuNC **2a** with BiAr_3_ **1a** was complete, the reaction mixture was filtered through a Celite pad. The filtrate was then treated with 1 N HCl aq., followed by the addition of *o*-phenylenediamine **8a**. The mixture was heated at 60 °C for 12 h to successfully afford the corresponding quinoxaline derivative **9a** in high yield. 4,5-Dimethyl-substituted *o*-phenylenediamine **8b** also reacted with the α-diimine **3aa** formed in situ to give the quinoxaline **9b** in high yield. In the case of *o*-phenylenediamine **8c**, which has an electron-withdrawing nitro group, the corresponding quinoxaline **9c** was formed in moderate yield. Moreover, when BiAr_3_ compounds with *p*-methyl- or *p*-fluoro-groups were employed for this cascade synthesis, the corresponding quinoxalines **9d** and **9e**, respectively, were obtained in good yields. The present method of quinoxaline synthesis is very convenient, because the *α*-diimines **3** formed in situ can be used directly without purification.

## 3. Materials and Methods

### 3.1. General Comments

All solvents were distilled before use. Triphenylbismuthine (**1a**) was purchased form a commercial source. The other bismuthines were prepared according to the literature. All aliphatic isocyanides and 2,6-xylylisocyanide (**2f**) were purchased from a commercial source. The other isocyanides were prepared according to the literature. *N*,*N’*-dialkyl α-diimines were isolated by recycle GPC (eluent: CHCl_3_). *N*,*N’*-diaryl α-diimines were isolated by preparative TLC (eluent: hexane/ethyl acetate). ^1^H NMR spectra were recorded on JEOL JNM-ECX400 (400 MHz) FT NMR or JEOL JNMECS400 (400 MHz) FT NMR in CDCl_3_ with Me_4_Si as an internal standard. ^13^C{^1^H} NMR spectra were recorded on JEOL JNM-ECX400 (100 MHz) FT NMR or JEOL JNM-ECS400 (100 MHz) FT NMR in CDCl_3_.

### 3.2. Typical Reaction Procedure for Ketimine Synthesis

In a dried 10 mL Schlenk test tube, norbornadiene rhodium(I) chloride dimer (0.04 mmol) and (*p*-MeOC_6_H_4_)_3_P (0.08 mmol) were dissolved in benzene (1.0 mL), and the mixture was stirred for 10 min at room temperature under Ar atmosphere. Then, triarylbismuthine (**1**; 0.4 mmol) and isocyanides (**2**; 0.4 mmol) were added to the reaction mixture. The resulting mixture was heated at 70 °C for 18 h. After the reaction, the crude product was filtered through a Celite pad using AcOMe as the eluent. All volatiles were evaporated under reduced pressure, and the yields of corresponding ketimines were determined by ^1^H NMR spectroscopy (solv.: CDCl_3_, internal standard: 1,3,5-trioxane) [43]. 

In this synthetic method, ketimine can be synthesized in a highly selective manner, and the purity is high even in the crude state. However, when treated with recycled GPC to remove unreacted starting substrates, the ketimine undergoes hydrolysis to produce a small amount of the corresponding ketone. Therefore, in order to use this ketimine synthesis method effectively, it is recommended to use it in one pot without isolating the ketimine. Ketimine **5da** and **5fa** could be isolated by recycled GPC (CH_2_Cl_2_), and their characterization data are shown as follows (^1^H and ^13^C{^1^H} NMR spectra are included in the Supplementary Materials):

*N*-*tert*-butyl-1,1-bis(4-(trifluoromethyl)phenyl)methanimine (**5da**). 43% yield (63.8 mg); white solid, m.p. 80.0–81.0 °C; ^1^H NMR (400 MHz, CDCl_3_) δ 7.70 (d, *J* = 8.2 Hz, 2H), 7.61 (d, *J* = 8.2 Hz, 2H), 7.54 (d, *J* = 8.6 Hz, 2H), 7.34 (d, *J* = 8.2 Hz, 2H), 1.17 (s, 9H); ^13^C{^1^H} NMR (100 MHz, CDCl_3_) δ 160.6, 144.3, 143.1, 131.5 (d, *J* = 32.6 Hz), 130.7 (d, *J* = 32.6 Hz), 130.3 (overlapped), 128.8, 128.3, 125.2 (d, *J* = 3.8 Hz), 125.1 (d, *J* = 3.9 Hz), 57.7, 31.6; HRMS (EI) m/z calcd for C_19_H_17_F_6_N [M]^+^: 373.1265, found: 373.1268.

*N*-*tert*-butyl-1,1-bis(4-chlorophenyl)methanimine (**5fa**) [CAS: 27126-15-4] [43]. 60% yield (73.0 mg); white solid, m.p. 104.0–105.0 °C; ^1^H NMR (400 MHz, CDCl_3_) δ 7.44 (d, *J* = 8.6 Hz, 2H), 7.39 (d, *J* = 8.6 Hz, 2H), 7.24 (d, *J* = 8.6 Hz, 2H), 7.12 (d, *J* = 8.2 Hz, 2H), 1.16 (s, 9H); ^13^C{^1^H} NMR (100 MHz, CDCl_3_) δ 161.2, 140.1, 137.8, 135.8, 134.2, 129.8, 129.4, 128.4, 128.2, 57.3, 31.6.

### 3.3. Typical Reaction Procedure for α-Diimine Synthesis (Schemes 3 and 4)

Triphenylbismuthine (**1a**; 0.4 mmol) and *tert*-butyl isocyanide (**2a**; 0.4 mmol) were dissolved in benzene (2 mL) in a dried two-necked test tube under a N_2_ atmosphere. Palladium diacetate (0.08 mmol) was added to the mixture. The resulting mixture was stirred for 18 h at 70 °C. After the reaction, the crude product was filtered through a Celite pad. All volatiles were evaporated under reduced pressure, and the NMR spectrum was measured (solv.: CDCl_3_). Dioxane was used as an internal standard. ^1^H and ^13^C{^1^H} NMR spectra are included in the Supplementary Materials.

*N*,*N*’-Bis(1,1-dimethylethyl)-1,2-diphenylethane-1,2-diimine (**3aa**) [CAS: 38015-77-9] [42]. 88% yield (56.3 mg); white solid; m.p. 107.5–109.0 °C; ^1^H NMR (400 MHz, CDCl_3_) δ 7.79–7.76 (m, 4H), 7.36–7.29 (m, 6H), 1.24 (s, 18H).

*N*,*N*’-Bis(1,1,3,3-tetramethylpropyl)-1,2-diphenylethane-1,2-diimine (**3ab**) [CAS: 1800598-80-4] [42]. 78% yield (67.4 mg); white solid; m.p. 108.0–109.0 °C; ^1^H NMR (400 MHz, CDCl_3_) δ 7.78–7.76 (m, 4H), 7.35–7.29 (m, 6H), 1.50 (s, 4H), 1.37 (s, 6H), 1.16 (s, 6H), 1.04 (s, 18H).

*N*,*N*’-Dicyclohexyl-1,2-diphenylethane-1,2-diimine (**3ac**) [CAS: 20586-41-8] [42]. 73% yield (54.3 mg); white solid; m.*p*. 89.5–91.0 °C; ^1^H NMR (400 MHz, CDCl_3_) δ 7.82–7.73 (m, 4H), 7.39–7.30 (m, 6H), 3.23 (tt, *J* = 9.6, 4.1 Hz, 2H), 1.90–1.82 (m, 2H), 1.75–1.17 (m, 16H), 1.15–0.98 (m, 2H).

*N*,*N*’-Dipentyl-1,2-diphenylethane-1,2-diimine (**3ad**) [CAS: 906560-91-6] [42]. 85% yield (59.2 mg); pale yellow oil; ^1^H NMR (400 MHz, CDCl_3_) δ 7.75–7.73 (m, 4H), 7.41–7.32 (m, 6H), 3.33 (t, *J* = 7.3 Hz, 4H), 1.73–1.65 (m, 4H), 1.36–1.24 (m, 8H), 0.86 (t, *J* = 7.3 Hz, 6H).

*N*,*N*’-Bis(4-methoxyphenyl)-1,2-diphenylethane-1,2-diimine (**3ae**) [CAS: 32349-49-8] [42]. 80% yield (33.6 mg); yellow solid; ^1^H NMR (400 MHz, CDCl_3_) δ 7.87–7.84 (m, 4H), 7.42–7.33 (m, 6H), 6.68–6.62 (m, 8H), 3.71 (s, 6H).

*N*,*N*’-Bis(1,1-dimethylethyl)-1,2-bis(4-methylphenyl)ethane-1,2-diimine (**3ba**) [CAS: 956375-94-3] [42]. 58% yield (40.4 mg); white solid; m.*p*. 83.5–85.0 °C; ^1^H NMR (400 MHz, CDCl_3_) δ 7.65 (d, *J* = 8.2 Hz, 4H), 7.11 (d, *J* = 8.2 Hz, 4H), 2.33 (s, 6H), 1.23 (s, 18H).

*N*,*N*’-Bis(1,1-dimethylethyl)-1,2-bis(4-fluorophenyl)ethane-1,2-diimine (**3ca**) [CAS: 884050-44-6] [42]. 27% yield (19.2 mg); white solid; m.*p*. 99.5–102.0 °C; ^1^H NMR (400 MHz, CDCl_3_) δ 7.77–7.72 (m, 4H), 7.03–6.98 (m, 4H), 1.23 (s, 18H).

*N*,*N*’-Bis(4-methoxyphenyl)-1,2-di-*p*-tolylethane-1,2-diimine (**3be**) [CAS: 86980-71-4] [44,45]. 47% yield (21.1 mg); ^1^H NMR (400 MHz, CDCl_3_) δ 7.75 (d, *J* = 8.2 Hz, 4H), 7.16 (d, *J* = 8.2 Hz, 4H), 6.62 (s, 8H), 3.72 (s, 6H), 2.36 (s, 6H); ^13^C{^1^H} NMR (100 MHz, CDCl_3_) δ 163.3, 157.1, 142.6, 141.1, 134.9, 129.4, 128.1, 122.1, 113.6, 55.3, 21.5.

*N*,*N*’-1,2-Tetrakis(4-methoxyphenyl)ethane-1,2-diimine (**3ee**) [CAS: 130440-66-3] [45]. 54% yield (25.9 mg); ^1^H NMR (400 MHz, CDCl_3_) δ 7.81 (d, *J* = 9.2 Hz, 4H), 6.87 (d, *J* = 9.2 Hz, 4H), 6.62 (s, 8H), 3.82 (s, 6H), 3.72 (s, 6H); ^13^C{^1^H} NMR (100 MHz, CDCl_3_) δ 162.8, 161.6, 157.0, 142.7, 130.4, 129.8, 122.1, 114.0, 113.6, 55.3.

### 3.4. Typical Reaction Procedure for Cascade Synthesis of 2,3-Diarylquinoxalines (Scheme 4)

Triphenylbismuthine (**1a**; 0.4 mmol) and *tert*-butyl isocyanide (**2a**; 1.2 mmol) were dissolved in MeCN (2 mL) in a dried two-necked test tube under air. Palladium diacetate (0.024 mmol) was added to the mixture. The resulting mixture was stirred for 4 h at 70 °C. After the reaction, the crude product was filtered through a Celite pad. All volatiles were evaporated under reduced pressure, after which dioxane (5 mL) and 1 N HCl (5 mL) were added. The mixture was stirred at 60 °C for 6 h, then α-diamine (0.6 mmol) was added and the reaction was stirred at 60 °C for 12 h. The mixture was extracted three times with EtOAc (10 mL), dried over MgSO_4_, and the desired product was purified by silica gel chromatography (eluent: hexane/EtOAc). ^1^H NMR spectra are included in the Supplementary Materials.

2,3-Diphenylquinoxaline (**9a**) [CAS: 1684-14-6] [46]. 84% yield (142.1 mg); white solid; m.*p*. 124–125 °C. ^1^H NMR (400 MHz, CDCl_3_) δ 8.20–8.16 (m, 2H), 7.79–7.75 (m, 2H), 7.53–7.51 (m, 4H), 7.39–7.31 (m, 6H).

6,7-Dimethyl-2,3-diphenylquinoxaline (**9b**) [CAS: 13362-56-6] [46]. 82% yield (152.5 mg); white solid; m.*p*. 174–175 °C; ^1^H NMR (400 MHz, CDCl_3_) δ 7.92 (s, 2H), 7.51–7.48 (m, 4H), 7.36–7.29 (m, 6H), 2.51 (s, 6H).

6-Nitro-2,3-diphenylquinoxaline (**9c**) [CAS: 7466-45-7] [46]. 55% yield (107.9 mg); yellow solid; m.*p*. 188–189 °C. ^1^H NMR (400 MHz, CDCl_3_) δ 9.08 (d, *J* = 2.7 Hz, 1H), 8.53 (dd, *J* = 9.1, 2.7 Hz, 1H), 8.30 (d, *J* = 9.5 Hz, 1H), 7.58–7.55 (m, 4H), 7.45–7.35 (m, 6H). 

2,3-di-*p*-tolylquinoxaline (**9d**) [CAS: 3719-84-4] [47]. 76% yield (141.4 mg); m.*p*. 142–144 °C; ^1^H NMR (400 MHz, CDCl_3_): δ 8.16–8.13 (m, 2H), 7.74–7.71 (m, 2H), 7.43 (d, *J* = 8.2 Hz, 4H), 7.14 (d, *J* = 8.2 Hz, 4H), 2.36 (s, 6H).

2,3-Bis(4-fluorophenyl)quinoxaline (**9e**) [CAS: 148186-43-0] [48]. 63% yield (120.2 mg); m.*p*. 134–136 °C; ^1^H NMR (400 MHz, CDCl_3_) δ 8.16 (dd, *J* = 6.3, 3.2 Hz, 2H), 7.78 (dd, *J* = 6.3, 3.6 Hz, 2H), 7.52–7.49 (m, 4H), 7.05 (t, *J* = 8.6 Hz, 4H).

## 4. Conclusions

In this study, we describe in detail the transition-metal-catalyzed diarylation of isocyanides with triarylbismuthines. When rhodium complexes were used as the catalyst in the diallylation reaction, ketimines (with one molecule of isocyanide incorporated) were highly selectively formed, whereas when palladium-based catalyst was used, *α*-diimines (with two molecules of isocyanide incorporated) were formed preferentially. For the purpose of further elucidating the details of this catalytic system, the effects of catalyst, solvent, and reaction temperature on the diarylation were investigated in detail to optimize the reaction conditions and determine the byproducts. Furthermore, the palladium-catalyzed diarylation was successfully applied to the cascade synthesis of quinoxalines via the formation of α-diimines as key intermediates.

## Figures and Tables

**Table 1:** Influence of reaction conditions on **5aa**/**3aa** selectivity.

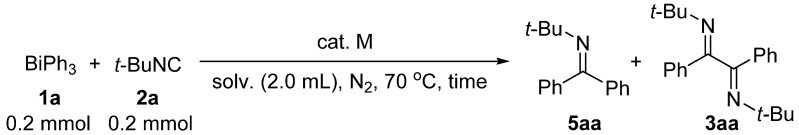					
Entry	Cat. M (mol%)	Solv.	Time (h)	Yields (%) ^a^	
				5aa	3aa
1	Pd(OAc)_2_ (20)	C_6_H_6_	18	9	90
2	Pd(PPh_3_)_4_ (20)	C_6_H_6_	18	trace	23
3	PdCl_2_ (20)	C_6_H_6_	18	5	71
4	Pd(PPh_3_)_2_Cl_2_ (20)	C_6_H_6_	18	11	62
5 ^b^	Pd(OAc)_2_ (20)	C_6_H_6_	18	5	66
6	Pd_2_(dba)_3_·CHCl_3_ (10)	C_6_H_6_	18	trace	56
7	none	C_6_H_6_	18	0	2
8 ^c^	Pd(OAc)_2_ (20)	C_6_H_6_	18	trace	58
9 ^d^	Pd(OAc)_2_ (20)	C_6_H_6_	18	10	82
10 ^e^	Pd(OAc)_2_ (20)	C_6_H_6_	18	8	59
11	Pd(OAc)_2_ (10)	C_6_H_6_	18	4	65
12	Pd(OAc)_2_ (5)	C_6_H_6_	18	4	51
13	Pd(OAc)_2_ (20)	THF	18	21	63
14	Pd(OAc)_2_ (20)	EtOH	18	trace	49
15	Pd(OAc)_2_ (20)	MeCN	18	0	84
16	Pd(OAc)_2_ (20)	PhMe	18	9	73
17	Pd(OAc)_2_ (20)	C_6_H_6_	4	1	81
18 ^f^	Pd(OAc)_2_ (20)	C_6_H_6_	18	6	77

^a^ Determined by ^1^H NMR. Calculated based on the amount of **2a**; ^b^ triphenylphosphine (40 mol%) was used as a ligand; ^c^ loading of **1a** was 0.1 mmol; ^d^ air; ^e^ room temp; ^f^ the reaction was conducted under Ar.

**Table 2:** Rh-catalyzed diarylation of *t*-BuNC with BiPh_3_.

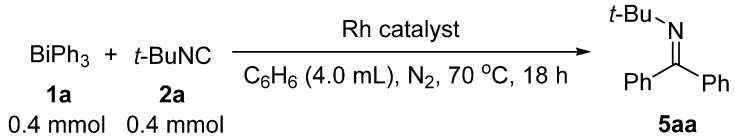		
Entry	Rh Catalyst (mol%)	Yield of 5aa (%) ^a^
1 ^b^	[RhCl(nbd)]_2_ (10)	50
2 ^b^	[RhCl(nbd)]_2_ (5)	32
3 ^b,c^	[RhCl(nbd)]_2_ (10)	75
4 ^b^	RhH(CO)(PPh_3_)_3_ (20)	20
5	[RhCl(nbd)]_2_/(*p*-MeO-C_6_H_4_)_3_P (10/20)	51
6 ^d^	[RhCl(nbd)]_2_/(*p*-MeO-C_6_H_4_)_3_P (10/20)	61
7 ^e^	[RhCl(nbd)]_2_/(*p*-MeO-C_6_H_4_)_3_P (10/20)	71
8 ^f^	[RhCl(nbd)]_2_/(*p*-MeO-C_6_H_4_)_3_P (10/20)	57
9 ^e,g^	[RhCl(nbd)]_2_/(*p*-MeO-C_6_H_4_)_3_P (10/20)	66
10	RhCl(PPh_3_)_3_ (10)	12
11	RhH(PPh_3_)_3_ (10)	12
12	RhBr(PPh_3_)_3_ (10)	17
13	*trans*-RhCl(CO)(PPh_3_)_3_ (10)	55
14	[Rh(dppp)(cod)]^+^BF_4_^−^ (10)	0
15	RhCl_3_ (10)	2
16	[Rh(OAc)_2_]_2_ (10)	6

^a^ Determined by ^1^H NMR. 1,3,5-Trioxane was used as an internal standard; ^b^ **1a** (0.2 mmol), *t*-BuNC (0.2 mmol), and benzene (2.0 mL) were used; ^c^ loading of **1a** was 0.4 mmol; ^d^ C_6_H_6_ (2.0 mL); ^e^ C_6_H_6_ (1.0 mL); ^f^ C_6_H_6_ (0.5 mL); ^g^ loading of **1a** was 0.6 mmol.

**Table 3:** Pd-catalyzed diarylation of *t*-BuNC with Bi(C_6_H_4_-F-*p*)_3_.

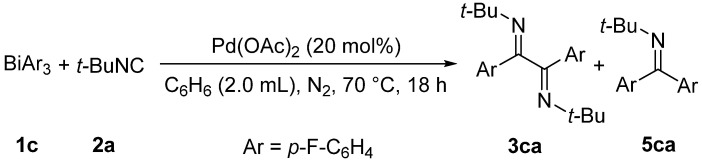				
Entry	2a (mmol)	1c (Equiv.)	Yields (%) ^a^	
			3ca	5ca
1	0.2	1.0	37	48
2	0.2	0.75	45	43
3	0.2	0.5	74	2

^a^ Determined by ^1^H NMR; calculated based on the amount of **2a**.

**Table 4:** Influence of oxidants on Pd-catalyzed diarylation of *t*-BuNC with BiPh_3_.

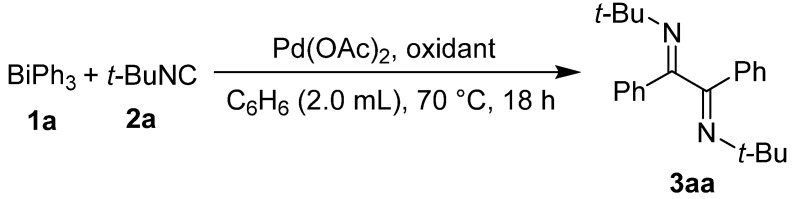					
Entry	Oxidant (Equiv.)	Cat. (mol%)	2a (mmol)	1a (Equiv.)	Yields (%) ^a^
1	none (N_2_ atm.)	5	0.4	1	27
2	air	5	0.4	1	85
3	O_2_	5	0.4	1	77
4	Cu(OAc)_2_∙H_2_O (1/2)	5	0.4	1	0
5	CuCO_3_∙Cu(OH)_2_∙H_2_O (1/4)	5	0.4	1	19
6	air	5	0.4	1/3	85 ^b^
7	air	1	1.5	1/3	69 ^b^
8	air	2	1.5	1/3	81 ^b^

^a^ Determined by ^1^H NMR. Calculated based on the amount of **2a**; ^b^ calculated based on the amount of the phenyl moieties on bismuth atom.

## Data Availability

The data presented in this study are available in the article and in its Supplementary Materials.

## Supplementary Materials

The following are available online at https://www.mdpi.com/article/10.3390/ma14154271/s1, Figure S1: Copies of ^1^H and ^13^C{^1^H} NMR spectra.

## References

[1] Marczenko K.M., Zurakowski J.A., Bamford K.L., MacMillan J.W.M., Chitnis S.S. (2019). Hydrostibination. Angew. Chem. Int. Ed..

[2] Chu T., Nikonov G.I. (2018). Oxidative addition and reductive elimination at main-group element centers. Chem. Rev..

[3] Dostál L. (2017). Quest for stable or masked pnictinidenes: Emerging and exciting class of group 15 compounds. Coord. Chem. Rev..

[4] Parke S.M., Boone M.P., Rivard E. (2016). Marriage of heavy main group elements with π-conjugated materials for optoelectronic applications. Chem. Commun..

[5] Yadav S., Saha S., Sen S.S. (2016). Compounds with Low-Valent p-Block Elements for Small Molecule Activation and Catalysis. ChemCatChem.

[6] Schulz S. (2015). Covalently bonded compounds of heavy group 15/16 elements—Synthesis, structure and potential application in material sciences. Coord. Chem. Rev..

[7] Xiong Y., Yao S., Driess M. (2013). Chemical Tricks to Stabilize Silanones and Their Heavier Homologues with E = O Bonds (E=Si−Pb): From Elusive Species to Isolable Building Blocks. Angew. Chem. Int. Ed..

[8] Sasamori T., Tokitoh N. (2013). A New Family of Multiple-Bond Compounds between Heavier Group 14 Elements. Bull. Chem. Soc. Jpn..

[9] Mandal S.K., Roesky H.W. (2012). Group 14 Hydrides with Low Valent Elements for Activation of Small Molecules. Acc. Chem. Res..

[10] Power P.P. (2011). Interaction of Multiple Bonded and Unsaturated Heavier Main Group Compounds with Hydrogen, Ammonia, Olefins, and Related Molecules. Acc. Chem. Res..

[11] Fischer R.C., Power P.P. (2010). π-Bonding and the lone pair effect in multiple bonds involving heavier main group elements: Developments in the new millennium. Chem. Rev..

[12] Escudié J., Ranaivonjatovo H. (2007). Group 14 and 15 Heteroallenes ECC and ECE’. Organometallics.

[13] Ollevier T. (2012). Bismuth-Mediated Organic Reactions.

[14] Gagnon A., Dansereau J., Le Roch A. (2017). Organobismuth Reagents: Synthesis, Properties and Applications in Organic Synthesis. Synthesis.

[15] Elliott G.I., Konopelski J.P. (2001). Arylation with organolead and organobismuth reagents. Tetrahedron.

[16] Finet J.-P. (1989). Arylation reactions with organobismuth reagents. Chem. Rev..

[17] Barton D.H.R., Ozbalik N., Ramesh M. (1988). The chemistry of organobismuth reagents. Part XIII ligand coupling induced by Pd(O). Tetrahedron.

[18] Barton D.H.R., Blazejewski J.-C., Charpiot B., Motherwell W.B. (1981). Tetraphenylbismuth Monofluoroacetate: A New Reagent for Regioselective Aryl Ether Formation. J. Chem. Soc. Chem. Commun..

[19] Barton D.H.R., Blazejewski J.-C., Charpiot B., Lester D.J., Motherwell W.G., Papoula M.T.B. (1980). Comparative Arylation Reactions with Pentaphenylbismuth and with Triphenylbismuth Carbonate. J. Chem. Soc. Chem. Commun..

[20] Abramovitch R.A., Barton D.H.R., Finet J.-P. (1988). Newer methods of arylation. Tetrahedron.

[21] Cho C.S., Yoshimori Y., Uemura S. (1995). Rhodium(I)- and Palladium(0)-Catalyzed Carbonylation of Triarylbismuthines with Carbon Monoxide via a Possible Oxidative Addition of a Carbon–Bismuth Bond to Rhodium(I) and Palladium(0). Bull. Chem. Soc. Jpn..

[22] Malysheva Y.B., Moiseev D.V., Gushchin A.V., Dodonov V.A. (2005). Palladium-Catalyzed C-Phenylation of Methyl Acrylate with Triphenylbismuth Dicarboxylates. Russ. J. Gen. Chem..

[23] Shimada S., Yamazaki O., Tanaka T., Rao M.L., Suzuki Y., Tanaka M. (2003). 5,6,7,12-tetrahydrodibenz[c,f][1,5]azabismocines: Highly reactive and recoverable organobismuth reagents for cross-coupling reactions with aryl bromides. Angew. Chem. Int. Ed..

[24] Qin W., Yasuike S., Kakusawa N., Sugawara Y., Kawahata M., Yamaguchi K., Kurita J. (2008). Triarylantimony dicarboxylates as pseudo-halides for palladium-catalyzed cross-coupling reaction with arylboronic acids and triarylbismuthanes without any base. J. Organomet. Chem..

[25] Rao M.L.N., Yamazaki O., Shimada S., Tanaka T., Suzuki Y., Tanaka M. (2001). Palladium-Catalyzed Cross-Coupling Reaction of Triarylbismuths with Aryl Halides and Triflates. Org. Lett..

[26] Murakami M., Masuda H., Kawano T., Nakamura H., Ito Y. (1991). Facile Synthesis of Vicinal Di- and Tricarbonyl Compounds by Samarium Diiodide-Mediated Double Insertion of Isocyanides into Organic Halides. J. Org. Chem..

[27] Onitsuka K., Ogawa K., Joh T., Takahashi S., Yamamoto Y., Yamazaki H. (1991). Reactions of *μ*-ethynediyl complexes of transition metals: Selective double insertion of isocyanides and molecular structure of [Cl(Et_3_P)_2_PdC≡CC(=NPh)C(=NPh)Pd(Et_3_P)_2_Cl]. J. Chem. Soc. Dalton Trans..

[28] Vicente J., Abad J.-A., Shaw K.F., Gil-Rubio J., Ramírez de Arellano M.C., Jones P.G. (1997). Palladium-Assisted Formation of Carbon−Carbon Bonds. 7.^1^ Reactions of (2,3,4-Trimethoxy-6-X-phenyl)palladium Complexes with Alkynes (X = C(O)NHBu^t^) and Isocyanides (X = C(O)NHBu^t^, C(O)Me, CHO): Crystal and Molecular Structures of [Pd{C_6_H{C(O)NHBu^t^}-6-(OMe)_3_-2,3,4}(bpy)](CF_3_SO_3_), [Pd{C(CO_2_Me)_=_C(CO_2_Me)C_6_H{C(O)NHBu^t^}-6-(OMe)_3_-2,3,4}-Cl(PPh_3_)], [Pd{C-(_=_NXy)C_6_H{C(O)NHBu^t^}-6-(OMe)_3_-2,3,4}-(bpy)](CF_3_SO_3_), and the Ketenimine 2-(2,6-Dimethylphenyl)-1-(((2,6-dimethylphenyl)imino)-methylene)-5,6,7-trimethoxy-3-oxoisoindoline. Organometallics.

[29] Vincente J., Abad J.-A., Frankland A.D., López-Serrano J., Ramírez de Arellano M.C., Jones P.G. (2002). Synthesis and Reactivity toward Isonitriles of (2-Aminoaryl)-palladium(II) Complexes. Organometallics.

[30] Owen G.R., Vilar R., White A.J.P., Williams D.J. (2003). Synthesis and Structural Characterization of a Novel Dipalladium Complex with an Unprecedented PdCN Bonding Motif. Organometallics.

[31] Dechert-Schmitt A.-M., Garnsey M.R., Wisniewska H.M., Murray J.I., Lee T., Kung D.W., Sach N., Blackmond D.G. (2019). Highly Modular Synthesis of 1,2- Diketones via Multicomponent Coupling Reactions of Isocyanides as CO Equivalents. ACS Catal..

[32] Peng X., Qin F., Xu M., Zhu S., Pan Y., Tang H., Meng X., Wang H. (2019). Synthesis of imidazo[1,2-c]thiazoles through Pd-catalyzed bicyclization of *tert*-butyl isonitrile with thioamides. Org. Biomol. Chem..

[33] Sun H., Tang S., Li D., Zhou Y., Huang J., Zhu Q. (2018). Cascade Double Isocyanide Insertion and C−N Coupling of 2-iodo-2’- isocyano-1,1’-biphenyls. Org. Biomol. Chem..

[34] Hu W., Li M., Jiang G., Wu W., Jiang H. (2018). Synthesis of 2,3-Difunctionalized Benzofuran Derivatives through Palladium-Catalyzed Double Isocyanide Insertion Reaction. Org. Lett..

[35] Hu W., Li J., Xu Y., Li J., Wu W., Liu H., Jiang H. (2017). Palladium-Catalyzed RedoxNeutral N−O/C(sp^3^)-H Functionalization of Aryl Oximes with Isocyanides. Org. Lett..

[36] Qiu G., Wang Q., Zhu J. (2017). Palladium-Catalyzed Three-Component Reaction of Propargyl Carbonates, Isocyanides, and Alcohols or Water: Switchable Synthesis of Pyrroles and Its Bicyclic Analogues. Org. Lett..

[37] Senadi G.C., Lu T.Y., Dhandabani G.K., Wang J.J. (2017). Palladium-Catalyzed Double-Isocyanide Insertion via Oxidative N−O Cleavage of Acetyl Oximes: Syntheses of 2*H*-Pyrrol-2-imines. Org. Lett..

[38] Chen Z.-B., Zhang Y., Yuan Q., Zhang F.-L., Zhu Y.-M., Shen J.-K. (2016). Palladium-Catalyzed Synthesis of α-Iminonitriles from Aryl Halides via Isocyanide Double Insertion Reaction. J. Org. Chem..

[39] Peng J., Gao Y., Hu W., Gao Y., Wu W., Ren Y., Jiang H. (2016). Palladium-Catalyzed Multicomponent Reaction (MCR) of Propargylic Carbonates with Isocyanides. Org. Lett..

[40] Senadi G.C., Hu W.-P., Boominathan S.S.K., Wang J.-J. (2015). Palladium(0)-Catalyzed Single and Double Isonitrile Insertion: A Facile Synthesis of Benzofurans, Indoles, and Isatins. Chem. Eur. J..

[41] Whitby R.J., Saluste C.G., Furber M. (2004). Synthesis of α-iminoimidates by palladium catalysed double isonitrile insertion. Org. Biomol. Chem..

[42] Kobiki Y., Kawaguchi S.-I., Ogawa A. (2015). Palladium-Catalyzed Synthesis of α-Diimines from Triarylbismuthines and Isocyanides. Org. Lett..

[43] Tran C.C., Kawaguchi S.-I., Kobiki Y., Matsubara H., Tran P.D., Kodama S., Nomoto A., Ogawa A. (2019). Palladium-Catalyzed Diarylation of Isocyanides with Tetraarylleads for the Selective Synthesis of Imines and α-Diimines. J. Org. Chem..

[44] Ōkubo M., Yoshida M., Horinouchi K., Nishida H., Fukuyama Y. (1983). Aryliminodimagnesium Reagents. VI. The Reactions with Conjugated Carbonyl Compounds. The Initial Coordination and the Electron-transfer in the Reactions of Organomagnesium Reagents. Bull. Chem. Soc. Jpn..

[45] Ōkubo M., Fukuyama Y., Sato M., Matsuo K., Kitahara T., Nakashima M. (1990). Reaction of aryliminodimagnesium with diphenyl diketones: Heat evolution accompanying single electron transfer and chelate formation, and product distribution. J. Phys. Org. Chem..

[46] Qi C., Jiang H., Huang L., Chen Z., Chen H. (2011). DABCO-Catalyzed Oxidation of Deoxybenzoins to Benzils with Air and One-Pot Synthesis of Quinoxalines. Synthesis.

[47] Akkilagunta V.K., Reddy V.P., Kakulapati R.R. (2010). Aqueous-Phase Aerobic Oxidation of Alcohols by Ru/C in the Presence of Cyclodextrin: One-Pot Biomimetic Approach to Quinoxaline Synthesis. Synlett.

[48] Karami B., Khodabakhshi S., Nikrooz M. (2011). Synthesis of Aza-Polycyclic Compounds: Novel Phenazines and Quinoxalines using Molybdate Sulfuric Acid (MSA). Polycyclic Aromat. Compd..

