# Supplementary material for: Transition-Metal-Catalyzed Diarylation of Isocyanides with Triarylbismuthines for the Selective Synthesis of Imine Derivatives
Source: Materials (Basel). 2021 Jul 30;14(15):4271. doi: 10.3390/ma14154271 (PMC8348920; doi:10.3390/ma14154271)
Supplement: Supplementary file 1 [file materials-14-04271-s001.zip › materials-1303300-supplementary.pdf]

materials

Supplementary Materials

Transition-Metal-Catalyzed Diarylation of Isocyanides with Triarylbismuthines for the Selective Synthesis of Imine Derivatives

Shintaro Kodama [1], Yuki Yamamoto [1], Yohsuke Kobiki [1], Hitomi Matsubara [1], Cong Chi Tran [1], Shin-ichi Kawaguchi [2,*], Akihiro Nomoto [1] and Akiya Ogawa [1,*]

[1] Department of Applied Chemistry, Graduate School of Engineering, Osaka Prefecture University, 1-1 Gakuen-cho, Nakaku, Sakai, Osaka 599-8570, Japan; skodama@chem.osakafu-u.ac.jp (S.K.); syb02137@edu.osakafu-u.ac.jp (Y.Y.); yohsuke.kobiki@gmail.com (Y.K.); sv108059@edu.osakafu-u.ac.jp (H.M.); mz105131@ edu.osakafu-u.ac.jp (C.C.T.); nomoto@chem.osakafu-u.ac.jp (A.N.)

[2] Center for Education and Research in Agricultural Innovation, Faculty of Agriculture, Saga University, 152-1 Shonan-cho, Karatsu, Saga 847-0021, Japan

* Correspondence: skawa@cc.saga-u.ac.jp (S.K.); ogawa@chem.osakafu-u.ac.jp (A.O.)

Citation: Kodama, S.; Yamamoto, Y.; Kobiki, Y.; Matsubara, H.; Tran, C.C.; Kawaguchi, S.-i.; Nomoto, A.; Ogawa, A. Transition-Metal-Catalyzed Diarylation of Isocyanides with Triarylbismuthines for the Selective Synthesis of Imine Derivatives. *Materials* **2021**, *14*, 4271. https://doi.org/10.3390/ma14154271

Academic Editor(s): Grzegorz Mlostoń

Received: 1 July 2021
Accepted: 28 July 2021
Published: 30 July 2021

Publisher's Note: MDPI stays neutral with regard to jurisdictional claims in published maps and institutional affiliations.

Copyright: © 2021 by the authors. Licensee MDPI, Basel, Switzerland. This article is an open access article distributed under the terms and conditions of the Creative Commons Attribution (CC BY) license (http://creativecommons.org/licenses/by/4.0/).

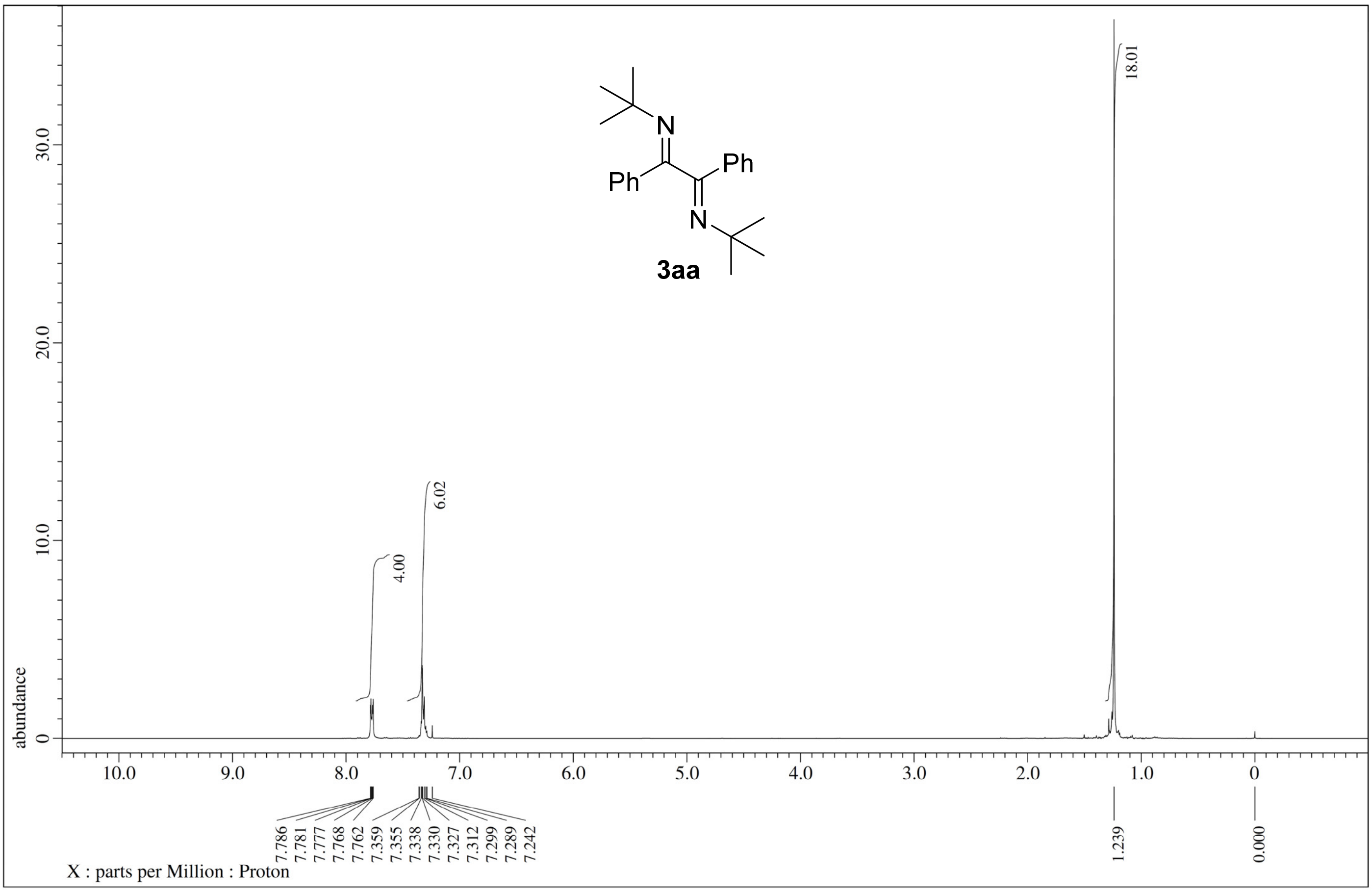
3aa
Ph
N
18.01
6.02
4.00
abundance
7.786
7.781
7.777
7.768
7.762
7.359
7.355
7.338
7.330
7.327
7.312
7.299
7.289
7.242
1.239
0.000
X : parts per Million : Proton

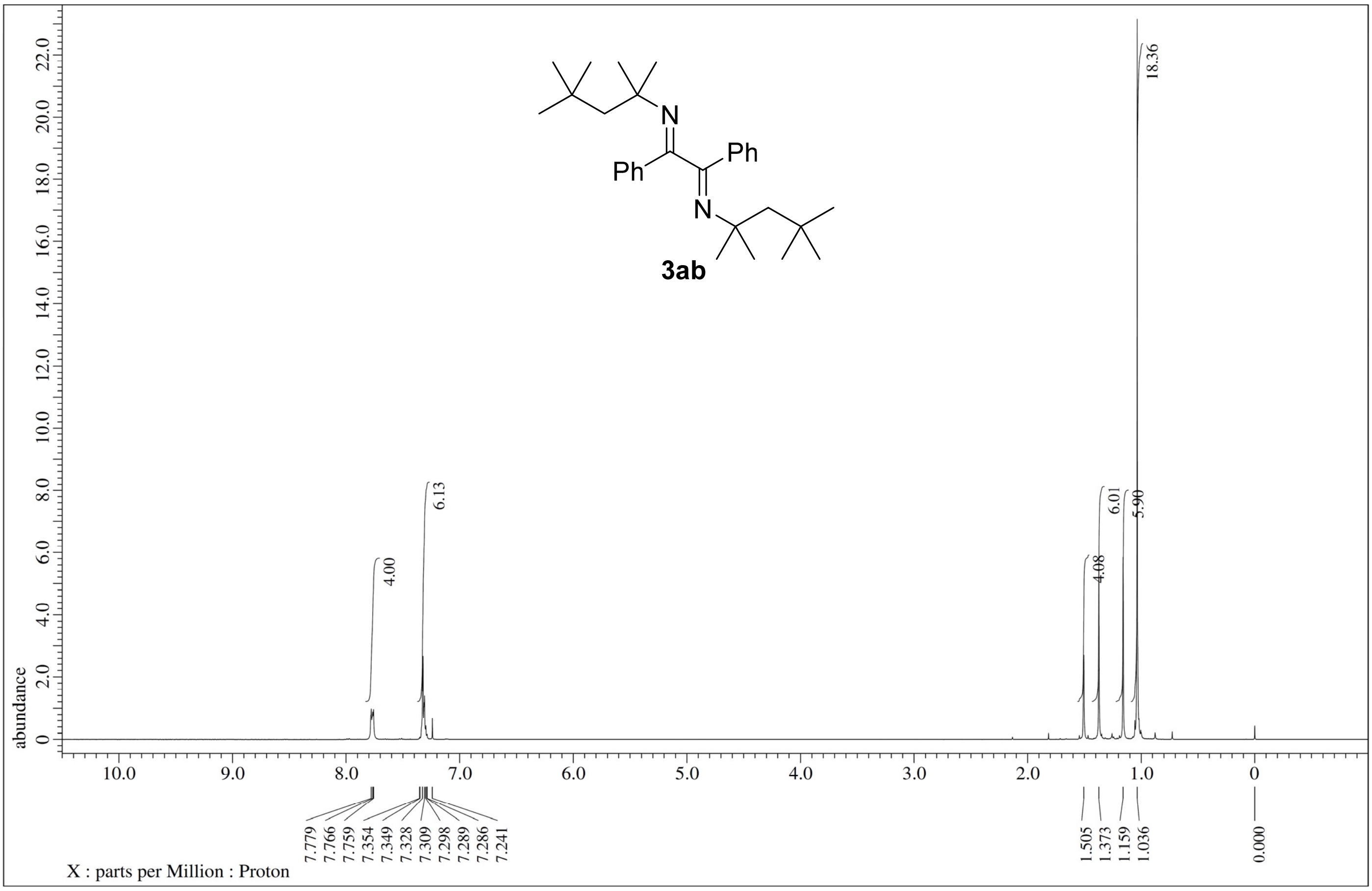
3ab
Ph
N
18.36
6.13
4.00
6.01
5.90
4.08
abundance
7.779
7.766
7.759
7.354
7.349
7.328
7.309
7.298
7.289
7.286
7.241
1.505
1.373
1.159
1.036
0.000
X : parts per Million : Proton

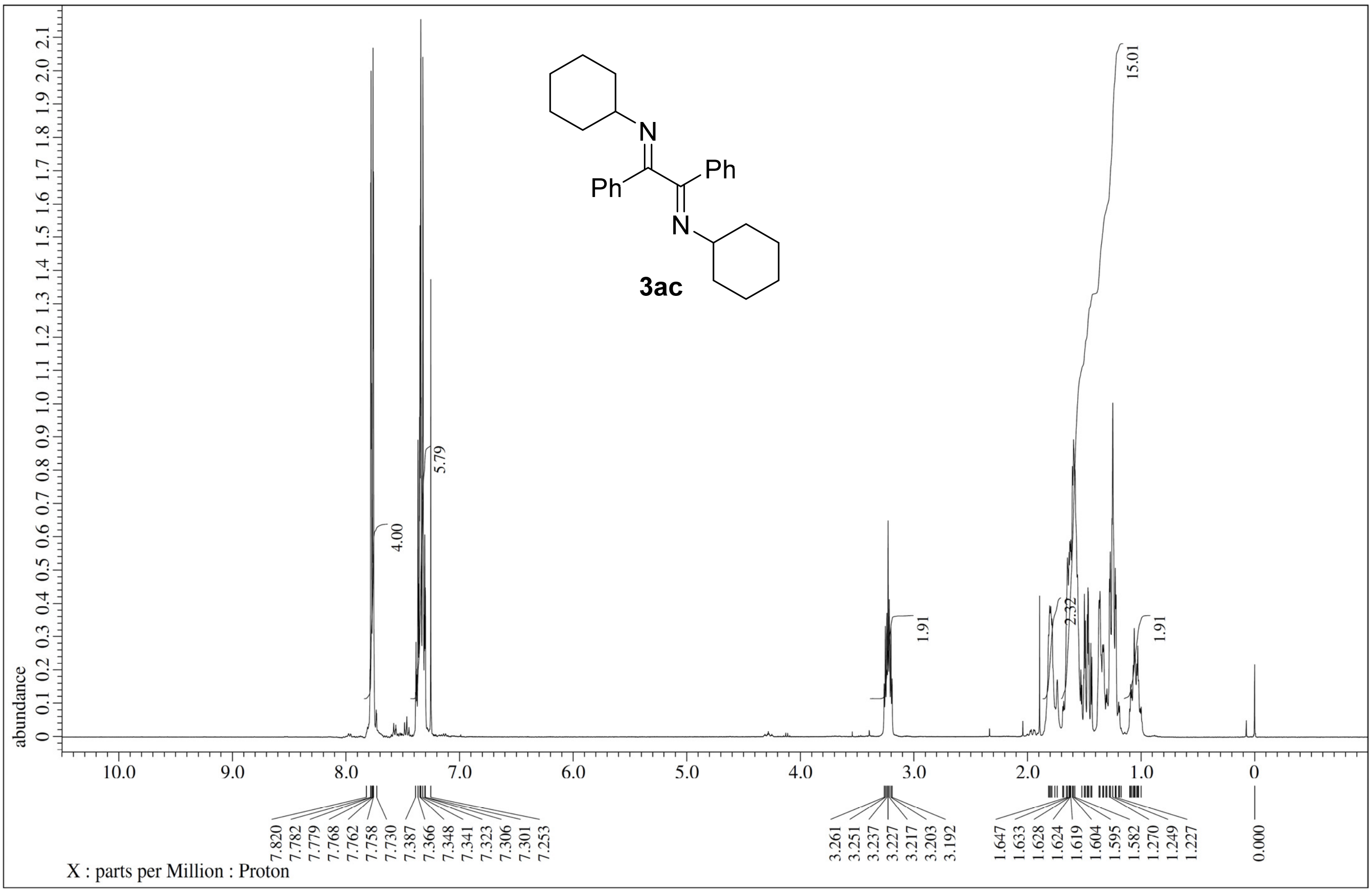
3ac
Ph
Ph
N
N
abundance
X : parts per Million : Proton
4.00
5.79
1.91
2.32
1.91
15.01

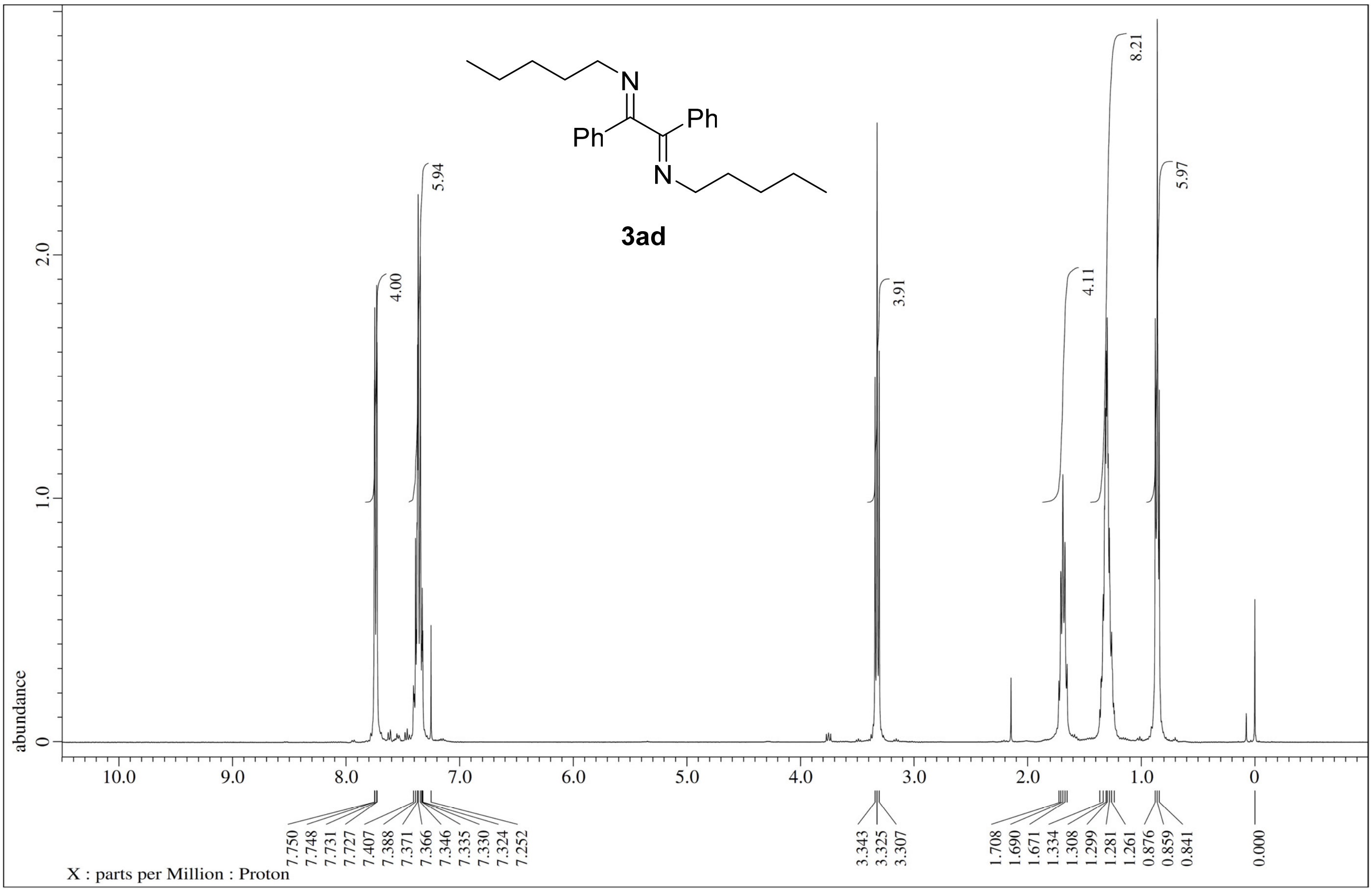
3ad
Ph
Ph
N
N
abundance
X : parts per Million : Proton
4.00
5.94
3.91
4.11
8.21
5.97

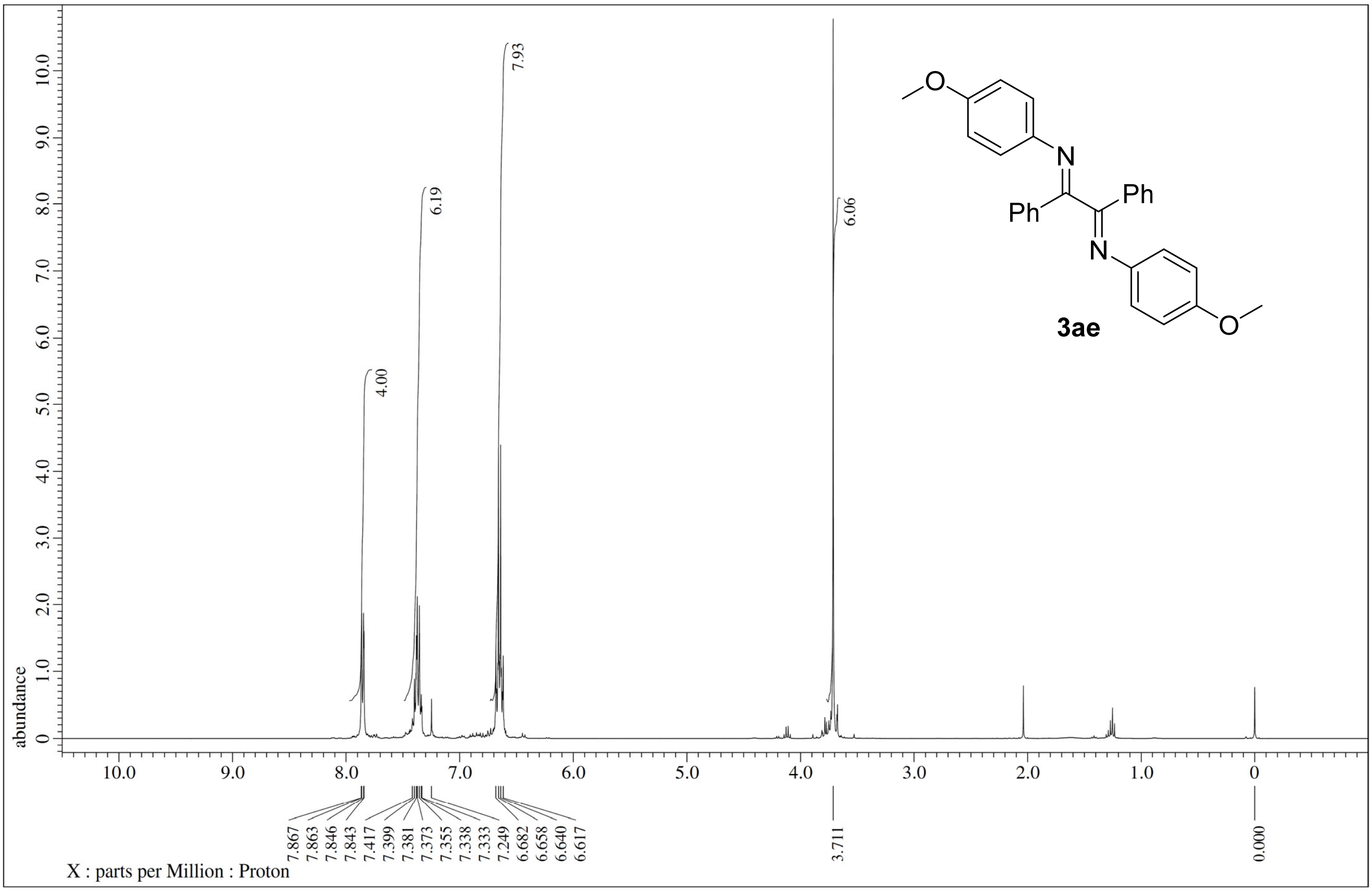

abundance
10.0
9.0
8.0
7.0
6.0
5.0
4.0
3.0
2.0
1.0
0
4.00
6.19
7.93
6.06
N
Ph
Ph
N
O
O
7.867
7.863
7.846
7.843
7.417
7.399
7.381
7.373
7.355
7.338
7.333
7.249
6.682
6.658
6.640
6.617
3.711
0.000
X : parts per Million : Proton

3ae

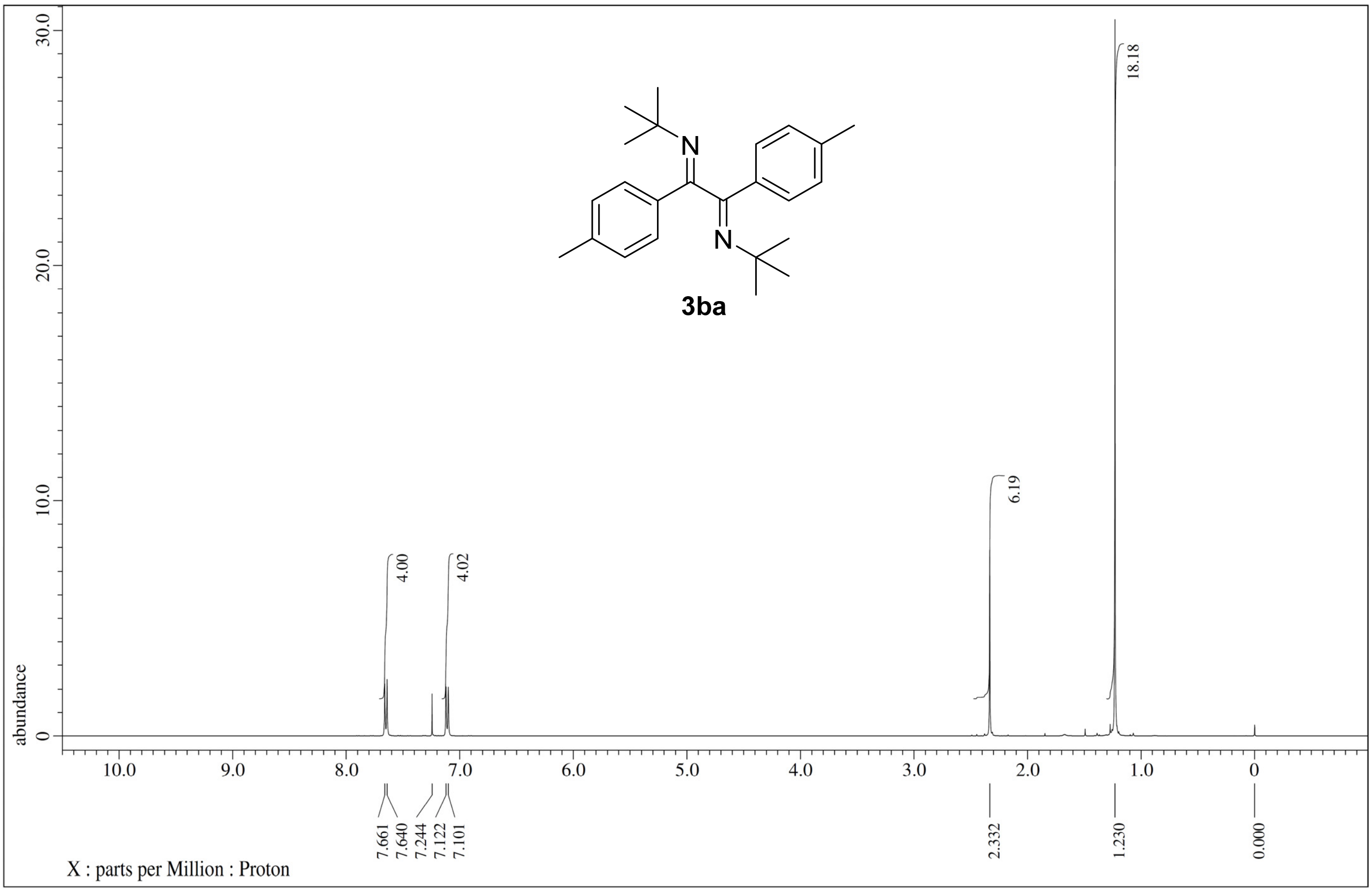

abundance
30.0
20.0
10.0
0
N
N
4.00
4.02
6.19
18.18
10.0
9.0
8.0
7.0
6.0
5.0
4.0
3.0
2.0
1.0
0
7.661
7.640
7.244
7.122
7.101
2.332
1.230
0.000
X : parts per Million : Proton

3ba

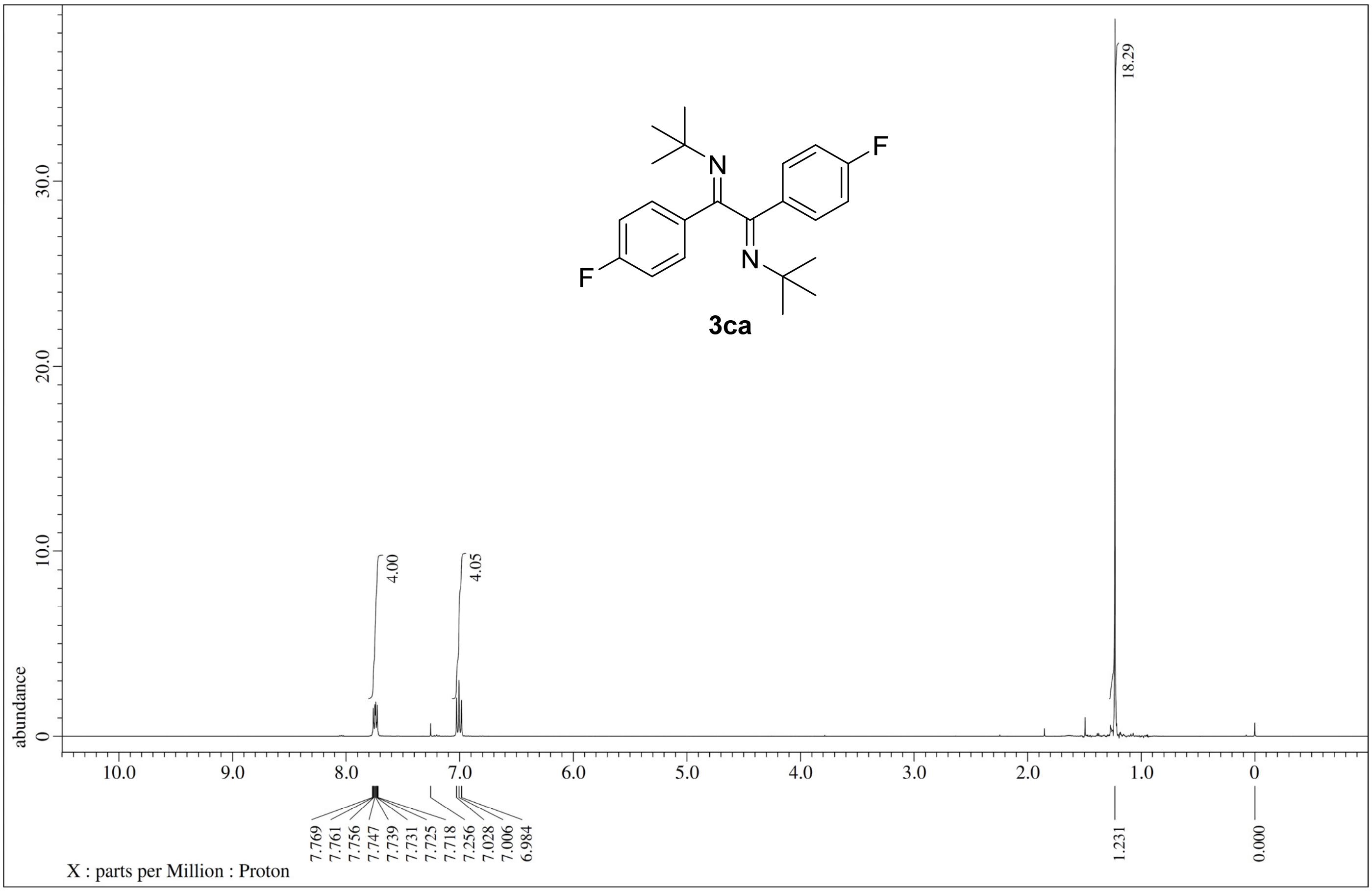
3ca
F
N
abundance
X : parts per Million : Proton
18.29
4.00
4.05
7.769 7.761 7.756 7.747 7.739 7.731 7.725 7.718 7.256 7.028 7.006 6.984
1.231
0.000

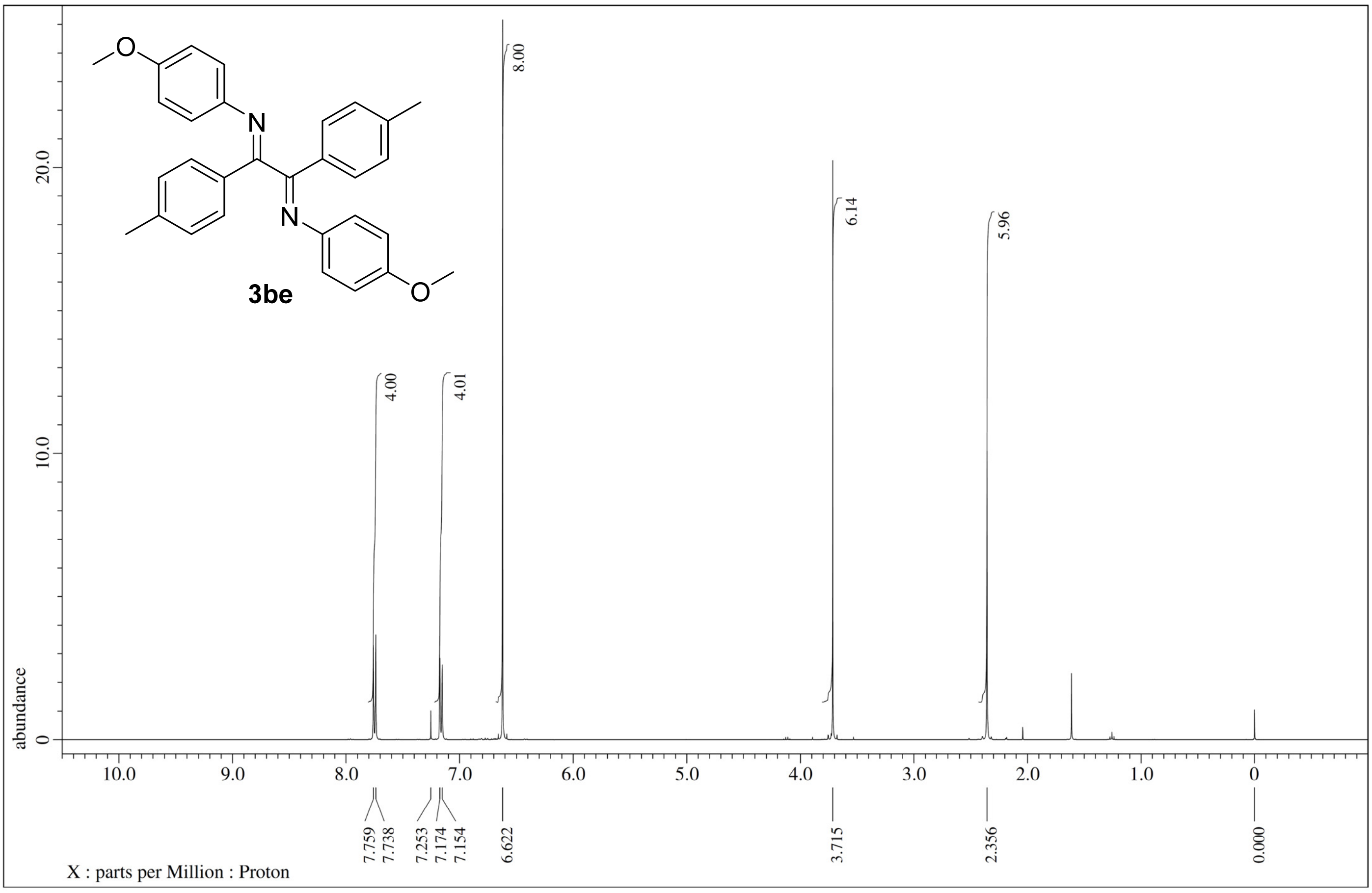
3be
O
N
abundance
X : parts per Million : Proton
8.00
6.14
5.96
4.00
4.01
7.759 7.738 7.253 7.174 7.154
6.622
3.715
2.356
0.000

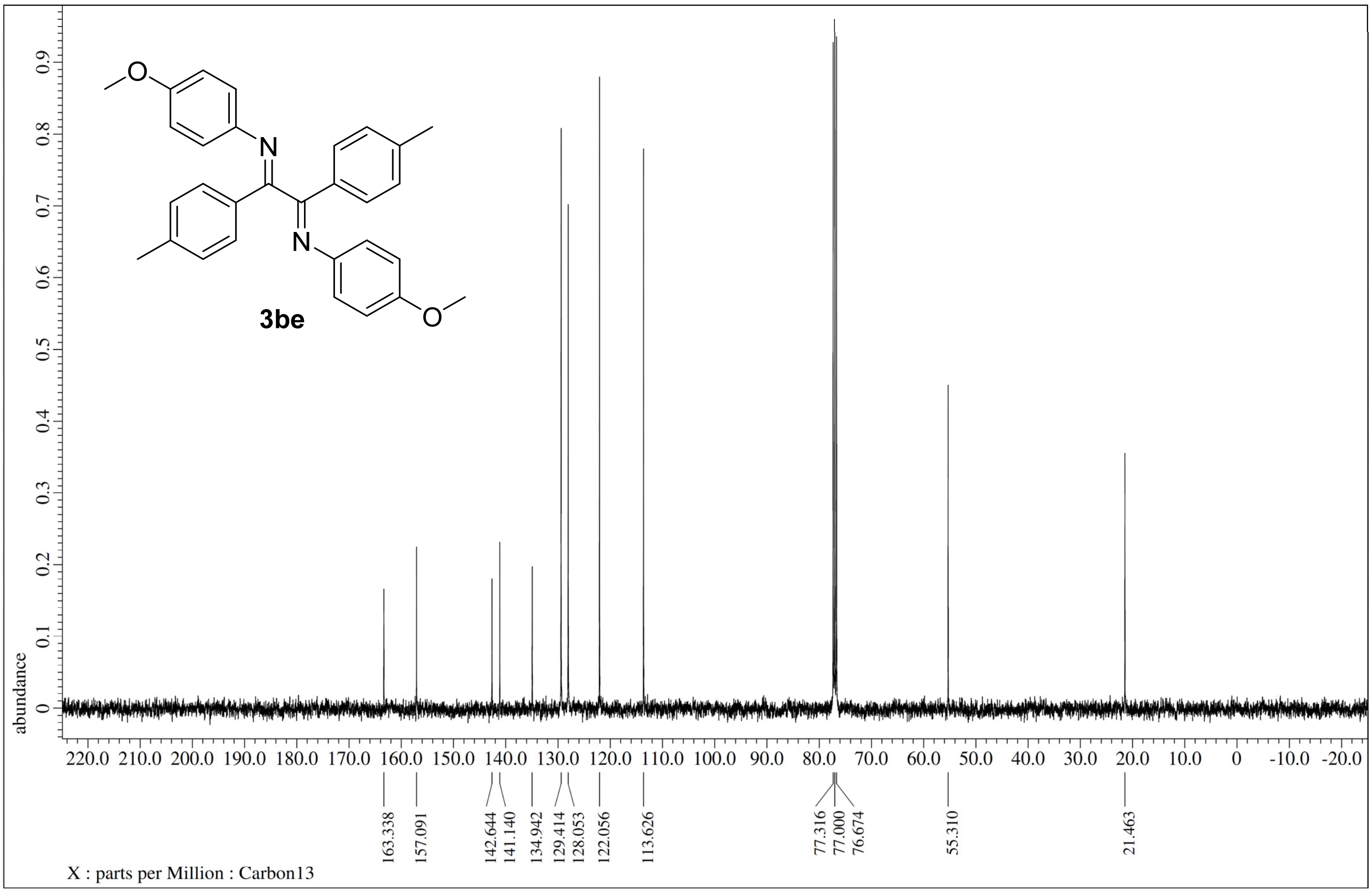
3be
abundance
163.338
157.091
142.644
141.140
134.942
129.414
128.053
122.056
113.626
77.316
77.000
76.674
55.310
21.463
X : parts per Million : Carbon13

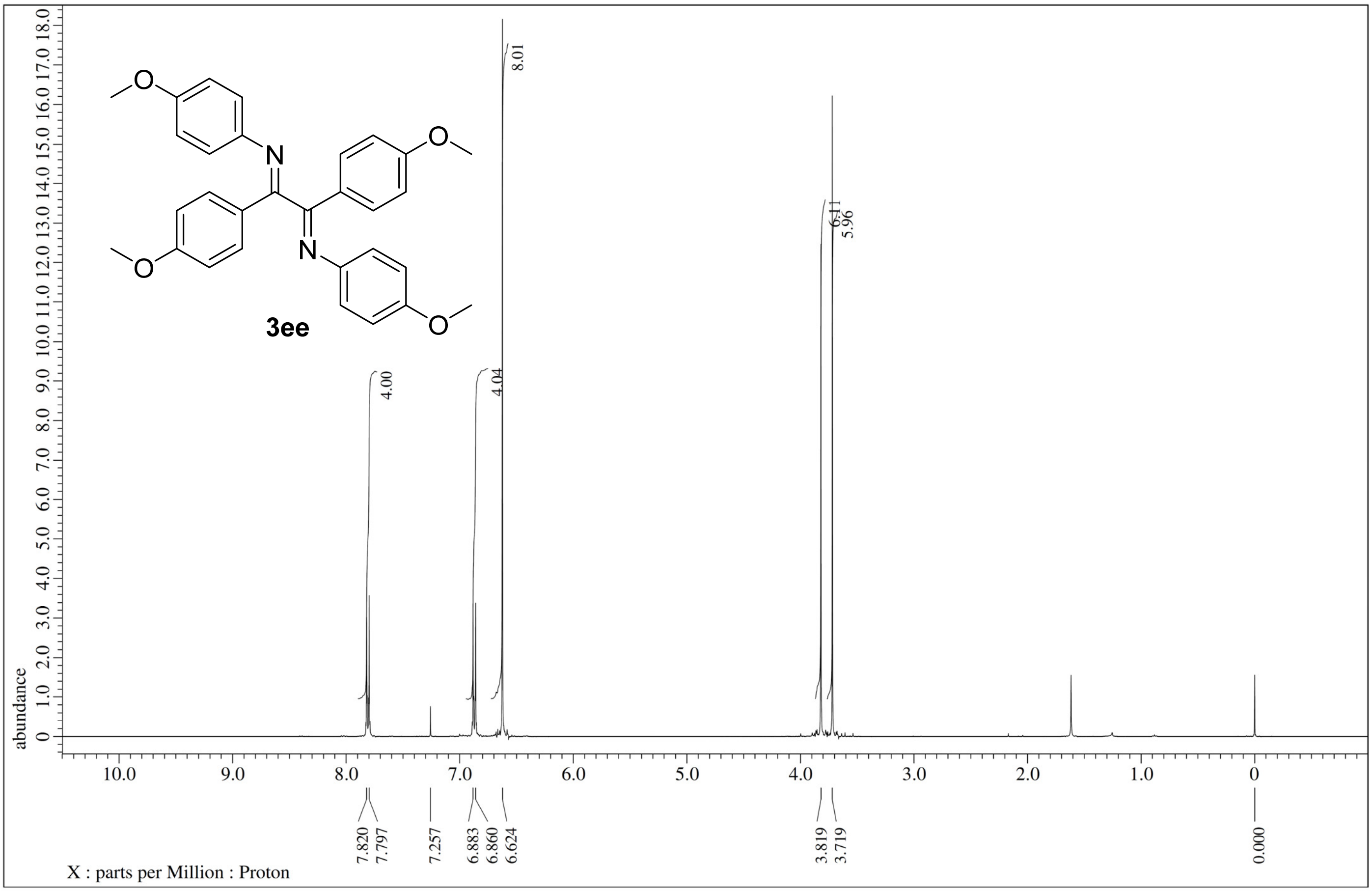
3ee
abundance
4.00
4.04
8.01
6.11
5.96
7.820
7.797
7.257
6.883
6.860
6.624
3.819
3.719
0.000
X : parts per Million : Proton

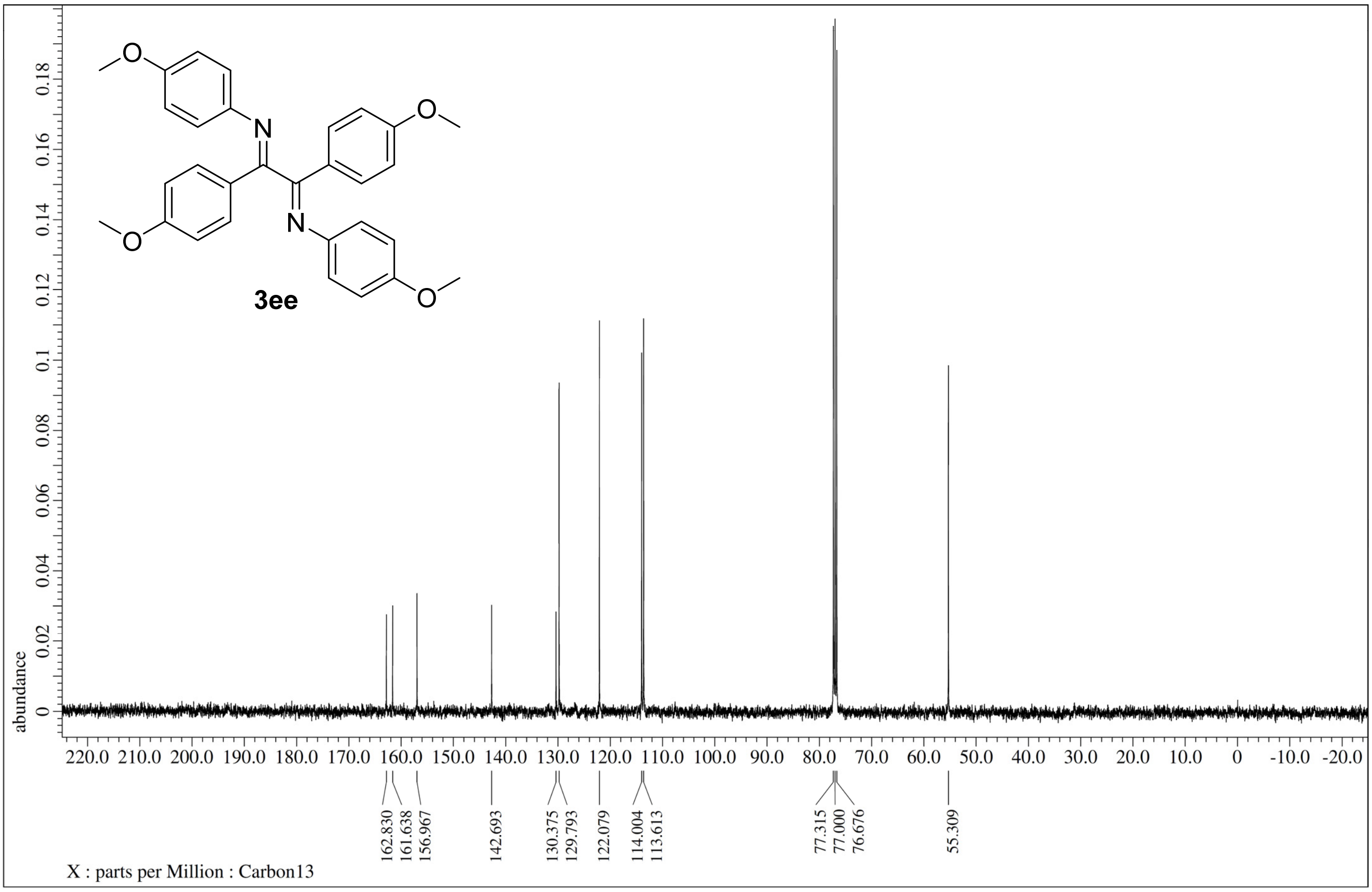
3ee
abundance
X : parts per Million : Carbon13
162.830
161.638
156.967
142.693
130.375
129.793
122.079
114.004
113.613
77.315
77.000
76.676
55.309

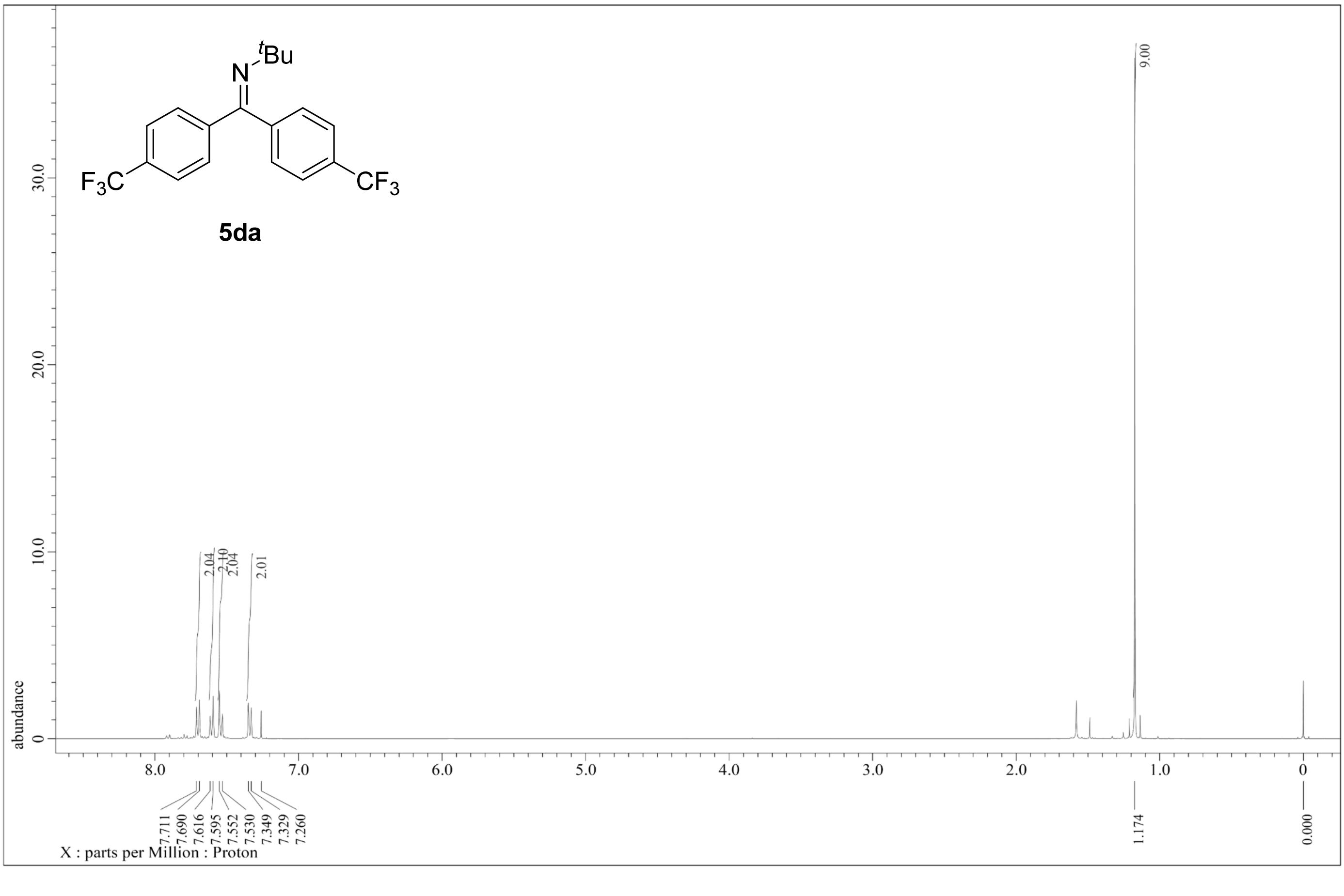
5da
abundance
X : parts per Million : Proton
7.711
7.690
7.616
7.595
7.552
7.530
7.349
7.329
7.260
1.174
0.000
9.00
2.04
2.10
2.04
2.01

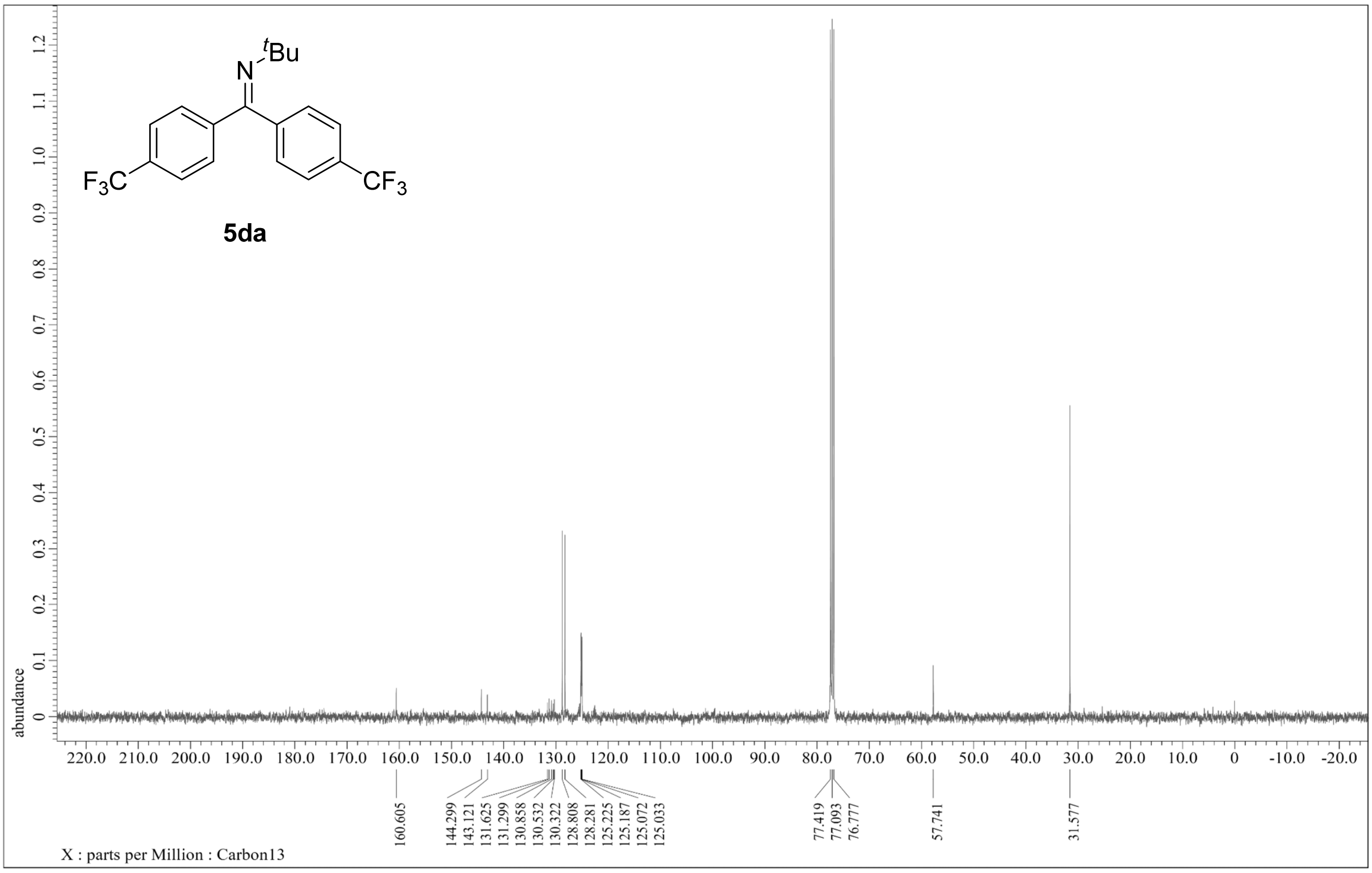

5da
abundance
X : parts per Million : Carbon13
160.605
144.299
143.121
131.625
131.299
130.858
130.532
130.322
128.808
128.281
125.225
125.187
125.072
125.033
77.419
77.093
76.777
57.741
31.577

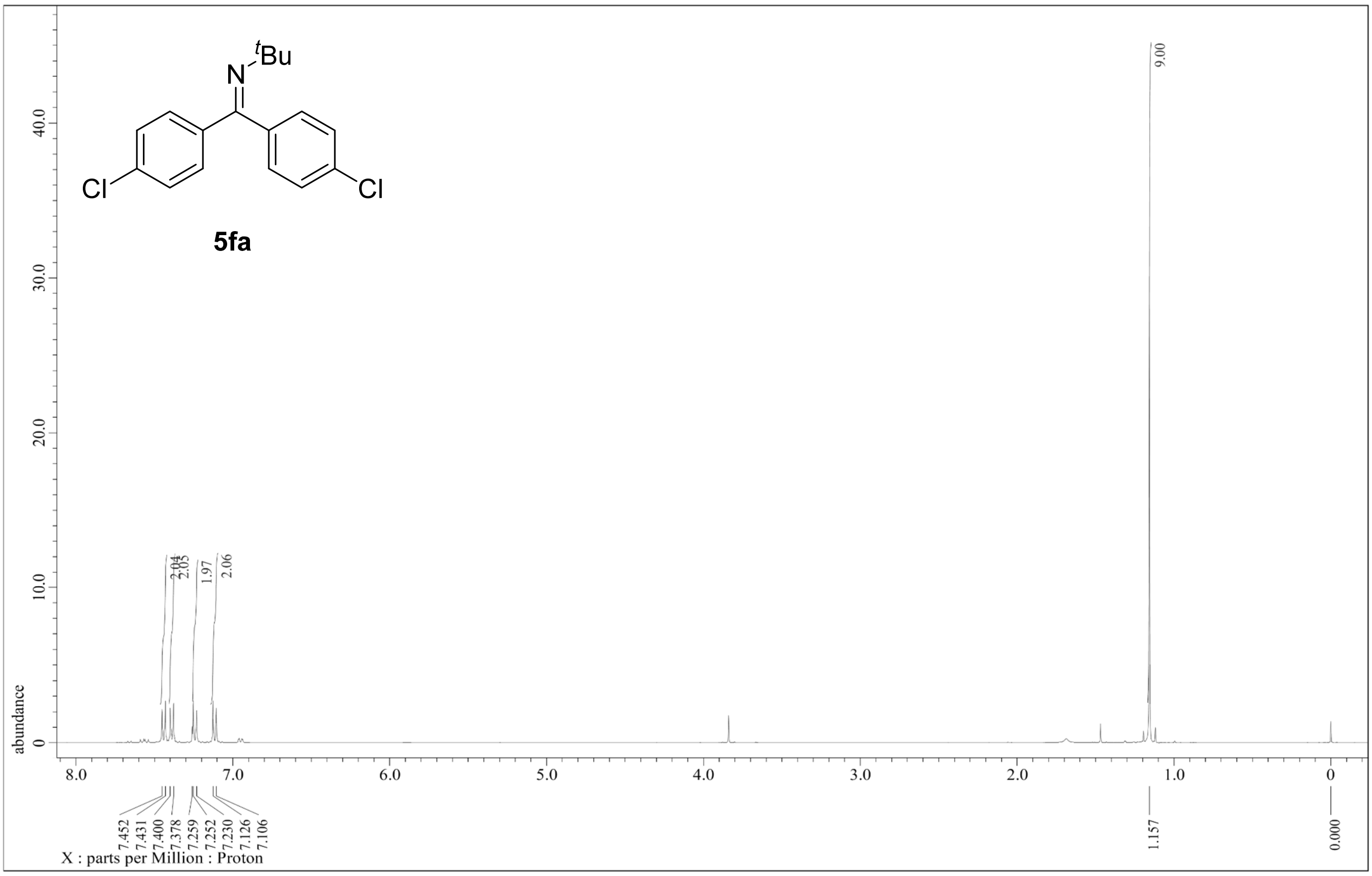

5fa
abundance
X : parts per Million : Proton
9.00
2.04
2.05
1.97
2.06
7.452
7.431
7.400
7.378
7.259
7.252
7.230
7.126
7.106
1.157
0.000

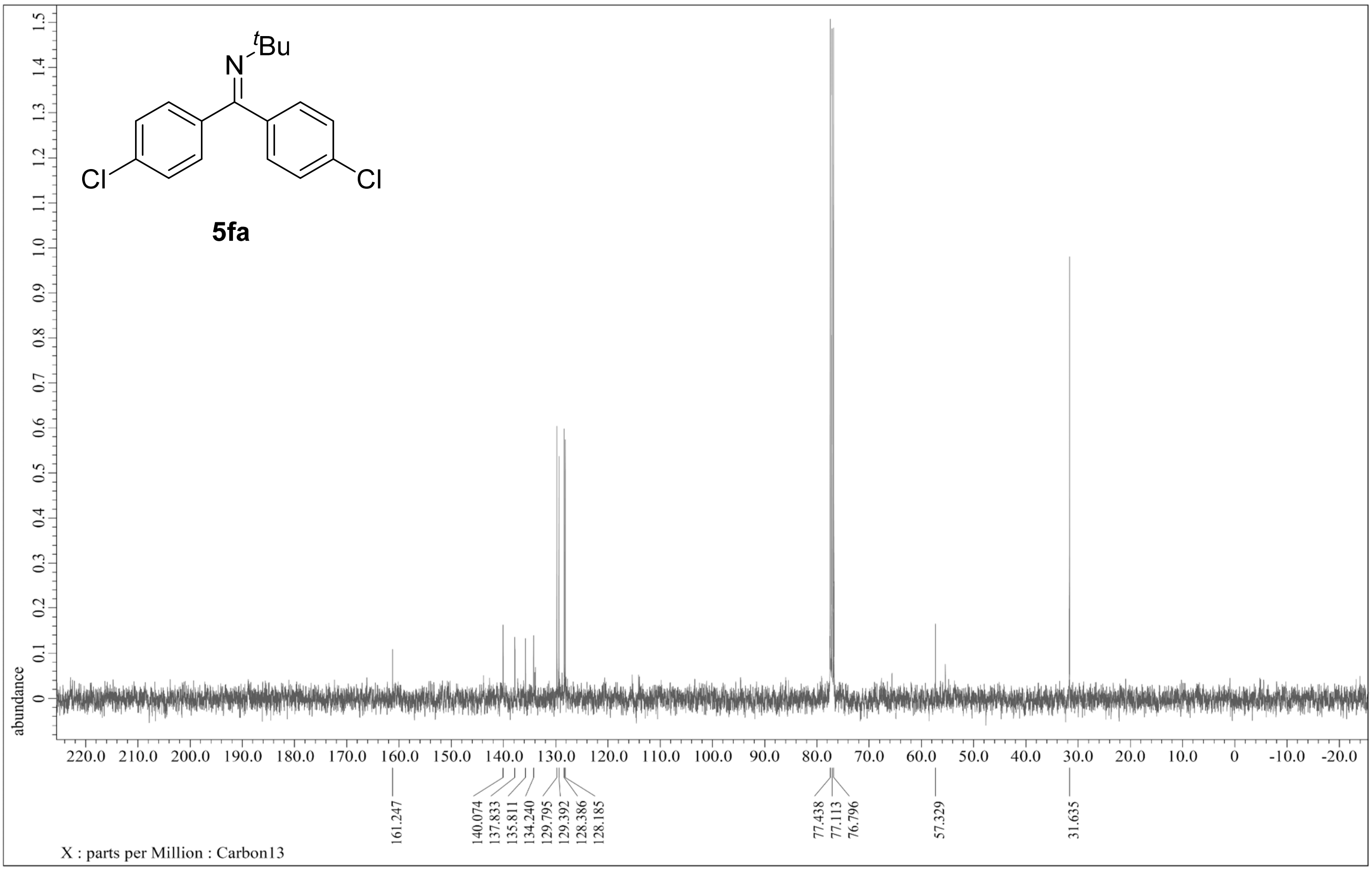

tBu
N
Cl
Cl
5fa
abundance
X : parts per Million : Carbon13
161.247
140.074
137.833
135.811
134.240
129.795
129.392
128.386
128.185
77.438
77.113
76.796
57.329
31.635

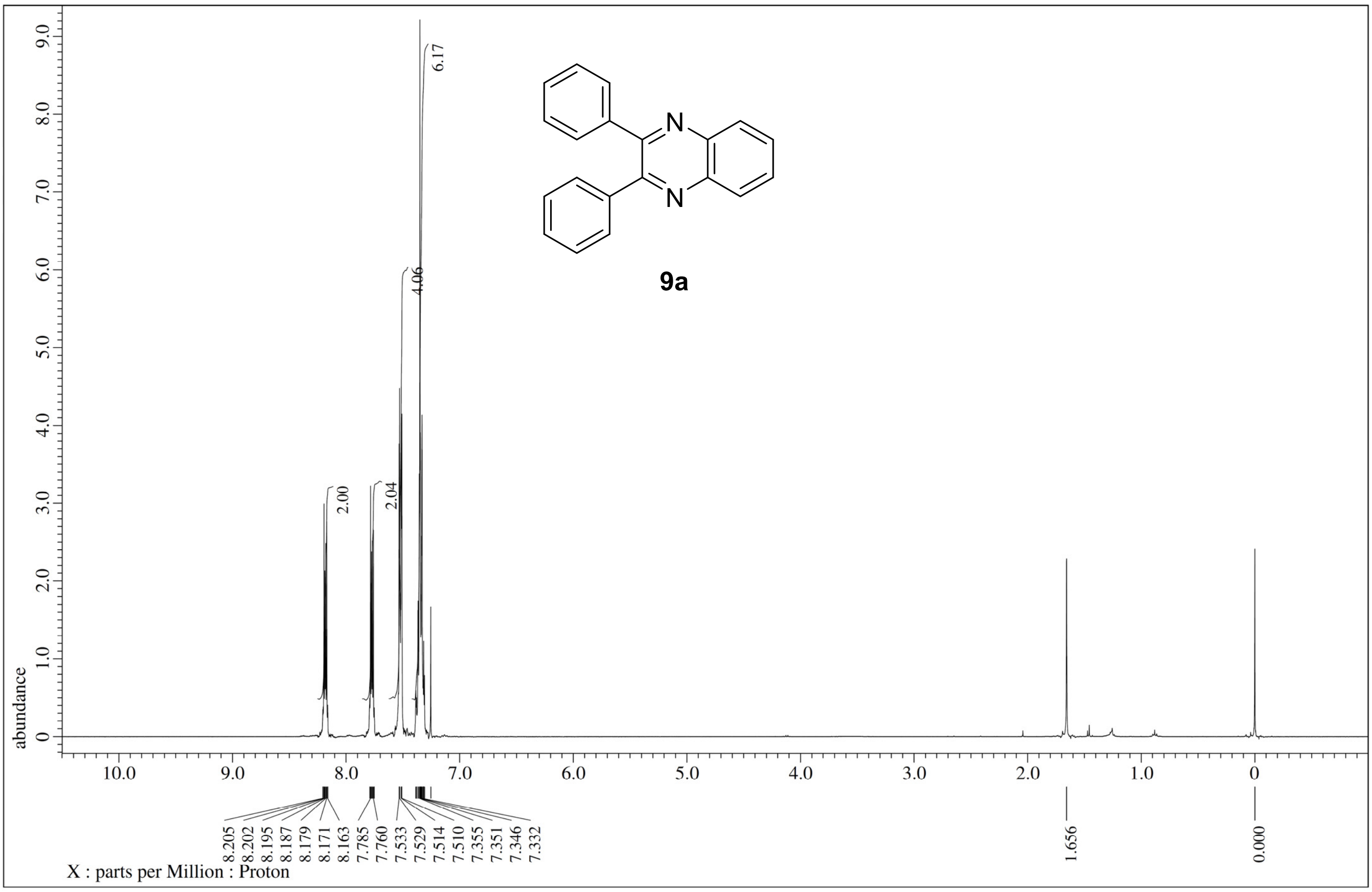

N
N
9a
abundance
X : parts per Million : Proton
2.00
2.04
4.06
6.17
8.205
8.202
8.195
8.187
8.179
8.171
8.163
7.785
7.760
7.533
7.529
7.514
7.510
7.355
7.351
7.346
7.332
1.656
0.000

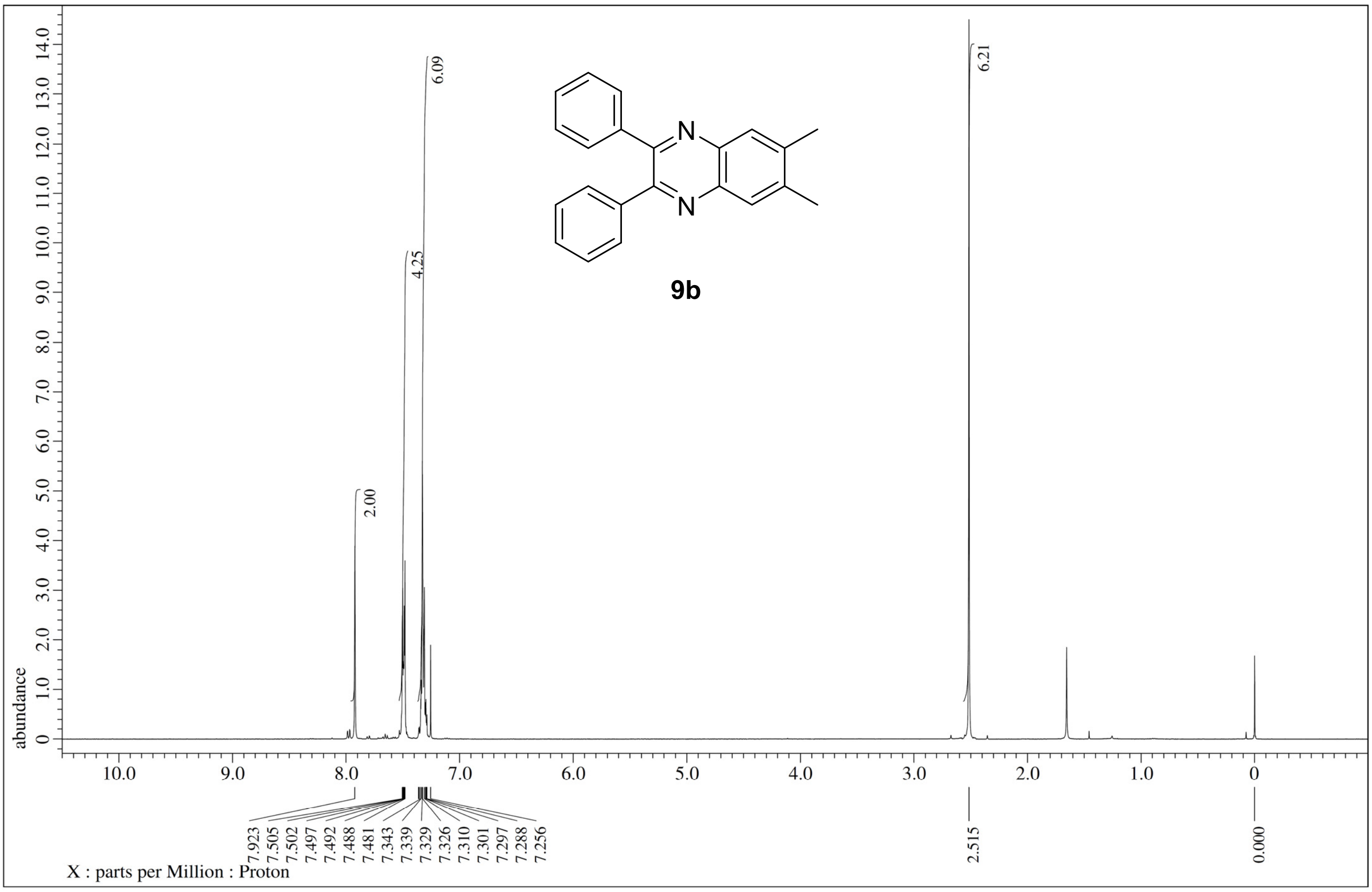
9b
N
N
abundance
X : parts per Million : Proton
2.00
4.25
6.09
6.21
7.923
7.505
7.502
7.497
7.492
7.488
7.481
7.343
7.339
7.329
7.326
7.310
7.301
7.297
7.288
7.256
2.515
0.000

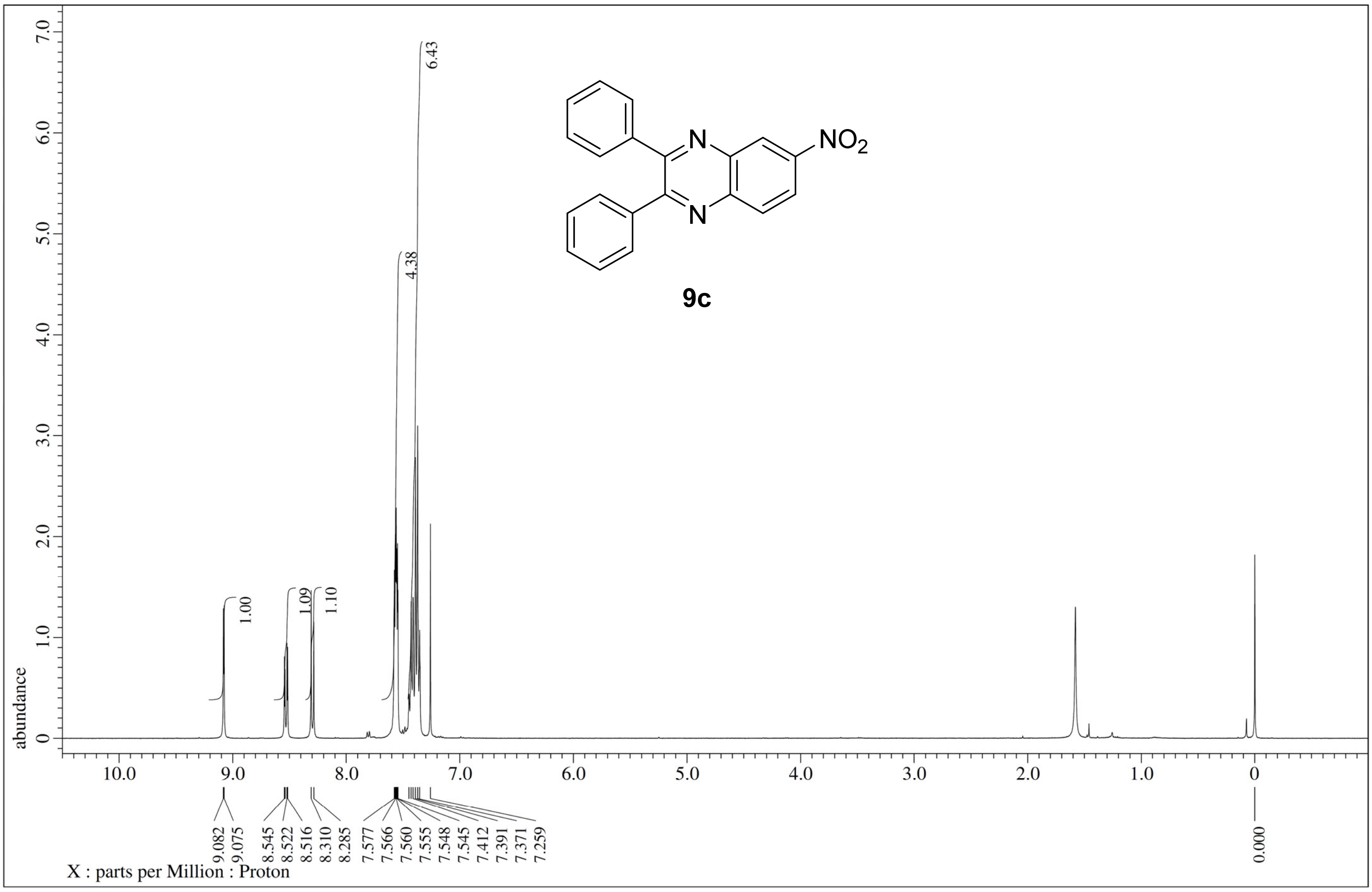
9c
N
N
NO2
abundance
X : parts per Million : Proton
1.00
1.09
1.10
4.38
6.43
9.082
9.075
8.545
8.522
8.516
8.310
8.285
7.577
7.566
7.560
7.555
7.548
7.545
7.412
7.391
7.371
7.259
0.000

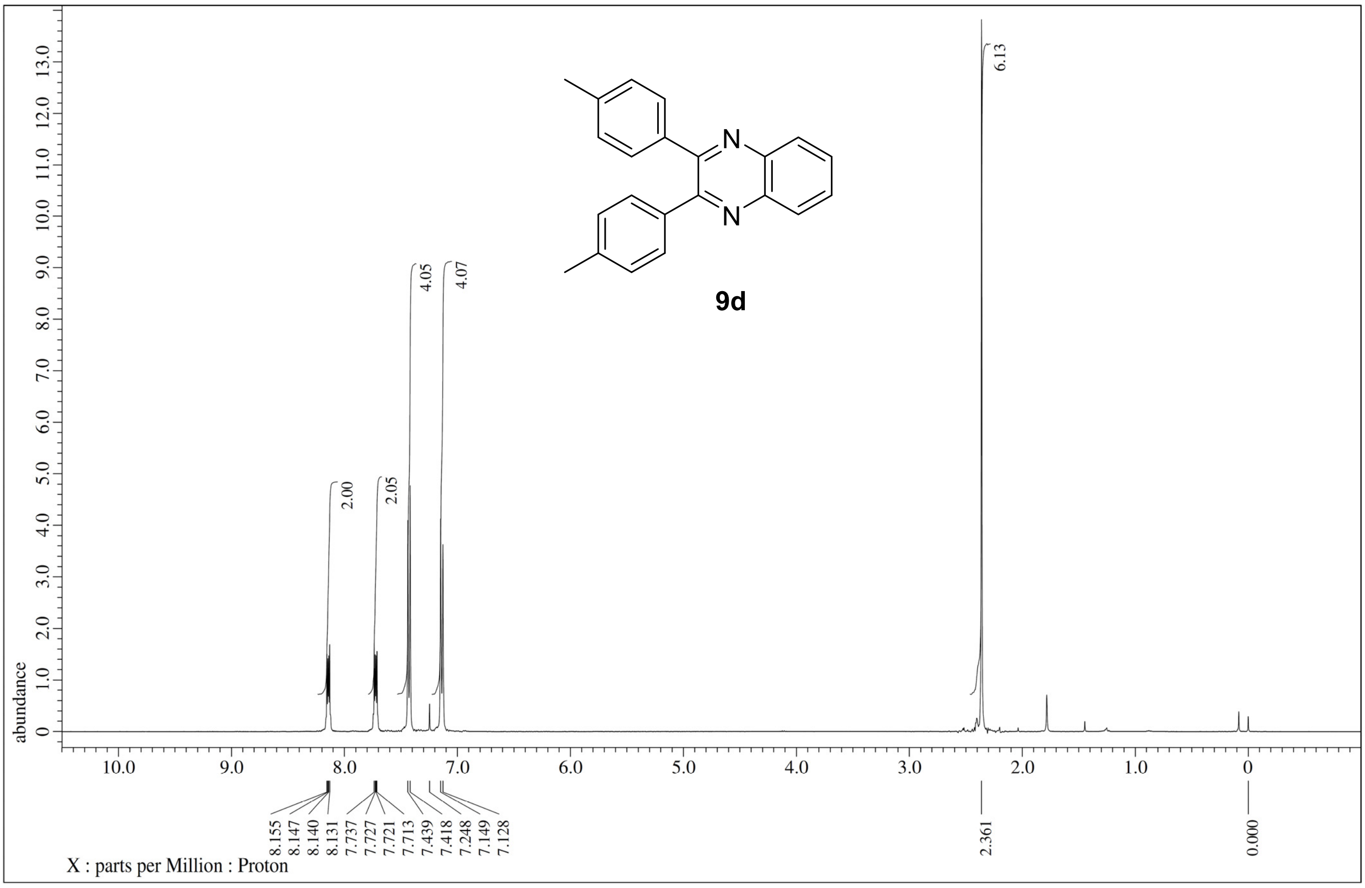

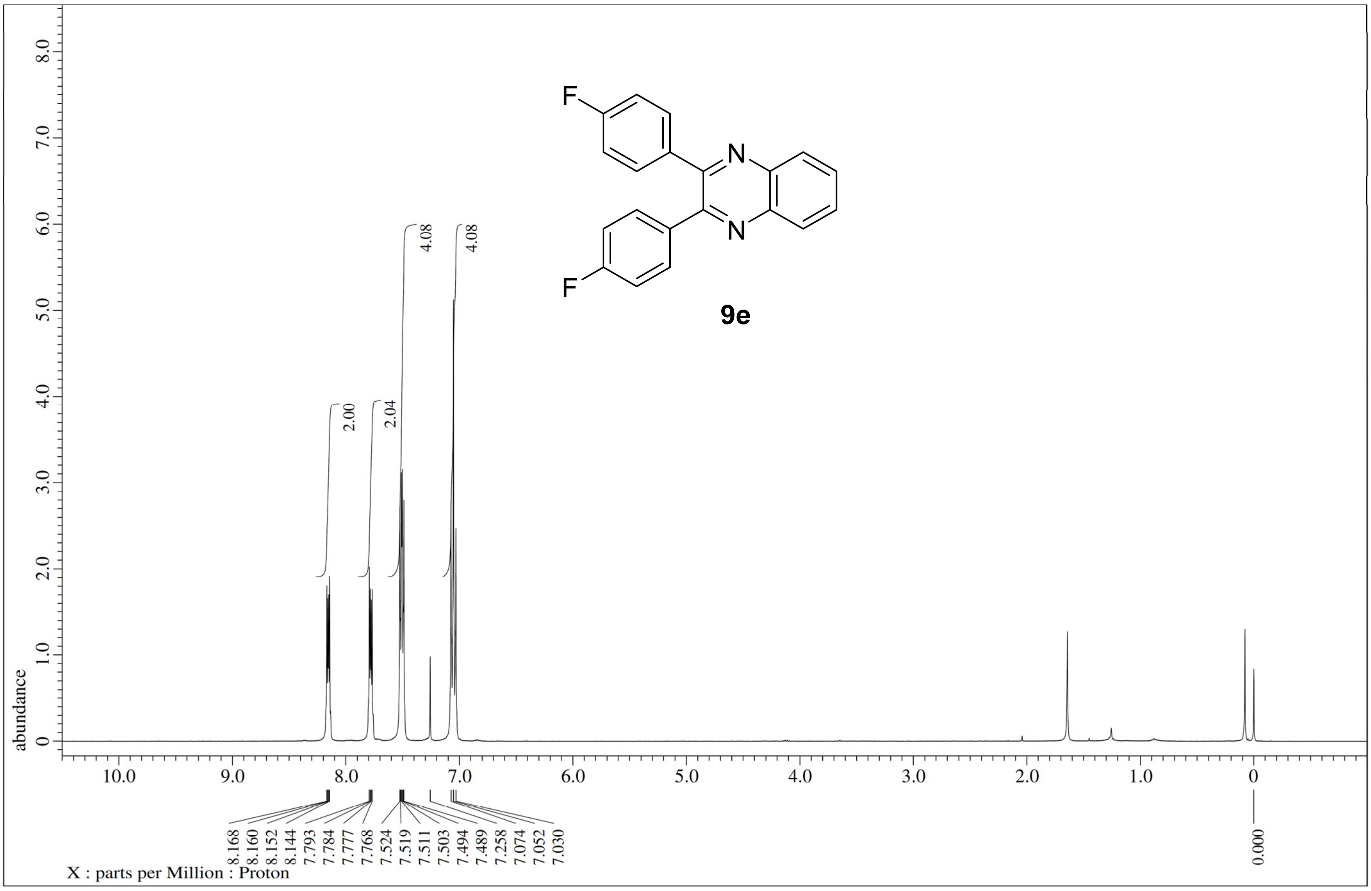

Figure S1. Copies of ^{1}H and ^{13}C{^{1}H} NMR spectra.
